# Compound to Extract to Formulation: a knowledge-transmitting approach for metabolites identification of Gegen-Qinlian Decoction, a traditional Chinese medicine formula

**DOI:** 10.1038/srep39534

**Published:** 2016-12-20

**Authors:** Xue Qiao, Qi Wang, Shuang Wang, Wen-juan Miao, Yan-jiao Li, Cheng Xiang, De-an Guo, Min Ye

**Affiliations:** 1 State Key Laboratory of Natural and Biomimetic Drugs, School of Pharmaceutical Sciences, Peking University, 38 Xueyuan Road, Beijing 100191, China

## Abstract

Herbal medicines usually contain a large group of chemical components, which may be transformed into more complex metabolites *in vivo*. In this study, we proposed a knowledge-transmitting strategy for metabolites identification of compound formulas. Gegen-Qinlian Decoction (GQD) is a classical formula in traditional Chinese medicine (TCM). It is widely used to treat diarrhea and diabetes in clinical practice. However, only tens of metabolites could be detected using conventional approaches. To comprehensively identify the metabolites of GQD, a “compound to extract to formulation” strategy was established in this study. The metabolic pathways of single representative constituents in GQD were studied, and the metabolic rules were transmitted to chemically similar compounds in herbal extracts. After screening diversified metabolites from herb extracts, the knowledge was summarized to identify the metabolites of GQD. Tandem mass spectrometry (MS^n^), fragment-based scan (NL, PRE), and selected reaction monitoring (SRM) were employed to identify, screen, and monitor the metabolites, respectively. Using this strategy, we detected 131 GQD metabolites (85 were newly generated) in rats biofluids. Among them, 112 metabolites could be detected when GQD was orally administered at a clinical dosage (12.5 g/kg). This strategy could be used for systematic metabolites identification of complex Chinese medicine formulas.

Traditional Chinese medicines (TCM) usually contain a large group of chemical components. These components may act synergistically to improve the therapeutic effects or independently to deal with different symptoms^1^,^2^. Meanwhile, phytochemicals and their *in vivo* metabolites may have comparative chances to exhibit the therapeutic effects^3^. Therefore, the metabolites could be critical to the bioactivities of TCM, and required unbiased elucidation.

Analytical chemists have made a lot of efforts to identify the metabolites of complex mixtures including TCM. Technologies including UPLC/SRM-MS and LC-NMR-MS significantly improved the detection sensitivity^4^,^5^, while several strategies emerged to predict and identify herbal metabolites globally. These strategies could be categorized into *chemistry-based* and *exposure-based*. The former one identified metabolites based on the chemistry of herbal medicine or prescription. For instance, Li *et al*. used seven LC/MS conditions to identify 28 catechols in DanHong Injection, and monitored their contents in volunteers^6^. Similarly, Wang *et al*. employed a UPLC-qTOF-MS/MS pharmacokinetics (PK) method to screen the absorbed components in Yin-Chen-Hao-Tang, a three-herb formula. They successfully monitored the PK of 21 compounds in rats plasma, and elucidated possible bioactive components by using hierarchical cluster analysis^7^. In these strategies, the biotransformed metabolites received less attention than the prototypes. The *exposure-based* strategies intended to systematically identify herbal compounds and their metabolites simultaneously. For instance, Gong *et al*. studied the metabolic network of TCM, and the possible relationship between original form and their metabolites^8^. Chen *et al*. identified 55 prototype compounds and 39 metabolites of Si-Ni Decoction *in vivo*^9^. Such strategies were unbiased, but might miss part of the metabolites.

Gegen-Qinlian Decoction (GQD) is a famous TCM formulation firstly recorded in the TCM ancient classic *Shang-Han-Lun* (Treatise on Febrile Diseases) of *Han* Dynasty (202 BC-220 AD). It is composed of four herbs, Puerariae Lobatae Radix (P), Scutellariae Radix (S), Coptidis Rhizoma (C), and Glycyrrhizae Radix et Rhizoma Praeparata cum Melle (G) in the ratio of 8:3:3:2 (*w/w/w/w*)^10^. According to the TCM formulation theory, P is the emperor herb of the formula playing the major therapeutic role. S and C are the minister herb, and G is the adjuvant and guide herb to harmonize the characteristics of other herbs to achieve optimal therapeutic effects and to reduce potential side effects. GQD is currently used in clinical practice to treat diarrhea^11^. More than 200 compounds have been reported from the four component herbs of GQD, thus far, including flavonoids (from P, S, and G), alkaloids (from C), and triterpenoid saponins (from G)^12^,^13^,^14^,^15^. Recently, We have identified 138 chemical constituents from GQD using 2D-LC/MS, and have determined the contents of 50 compounds in GQD and its patent drugs^16^,^17^. However, only a few reports are available on the metabolism of GQD. Hou *et al*. monitored the plasma concentrations of 10 phytochemicals and 6 phase II metabolites for decoction and concentrated powder of GQD^18^. We had also investigated the metabolites of GQD, and identified 42 *in vivo* metabolites (21 of them were detected in plasma)^19^. The *in vivo* exposed metabolites of GQD were not systematically characterized given its complex chemical composition.

In our previous report, we had developed a “compound to extract” strategy to comprehensively characterize the metabolites of licorice water extract^20^, and monitored the PK of 55 licorice compounds and metabolites in rats^21^. It used the metabolic route of representative single compounds to predict and identify the metabolites of other compounds. In the present study, we intend to improve this strategy so that it is compatible with the more complex formula GQD. As depicted in Fig. 1, firstly, we use tandem mass spectrometry (MS^n^) to study the metabolic rules of 19 representative compounds (eight groups with different scaffolds). Secondly, we use neutral loss scan (NL) and precursor ion scan (PRE) of LC/MS/MS analysis to systematically characterize the metabolites of each component herb. Finally, the identified metabolites are confirmed by qTOF-MS, and then detected by the sensitive SRM scan mode of LC/MS/MS. By following this strategy, we detected 131 metabolites (including 46 original phytochemicals and 85 newly formed ones) in rats after a single dose oral administration of GQD. Among them, 112 metabolites could be detected at a clinical dosage (12.5 g/kg). Furthermore, parent compounds for the detected metabolites were interpreted.

## Results

### Metabolic pathways of representative single compounds

In our previous study, we selected 10 representative compounds from licorice, elucidated their metabolic rules, and used these rules for metabolite identification of other compounds which have the similar scaffolds in licorice extract, developing a “compound to extract” strategy^20^. In the present study, this strategy was extrapolated to the four-herb formulation GQD. Chemical constituents in GQD could be classified into eight groups according to their structural type: flavonoid *C*-glycosides (*A*), flavonoid *O*-glucuronides (*B*), benzylisoquinoline alkaloids (*C*), free flavonoids (*D*), flavonoid *O*-glycosides (*E*), coumarins (*F*), triterpenoid saponins (*G*), and other atypical and abundant backbones (*H*). Among them, groups *D, E, F* and *G* had been investigated in our previous studies on licorice^20^,^21^. The metabolic pathways of flavonoid *C*-glycosides, *O*-glucuronides, and alkaloids were reported in this paper. In total, 19 single compounds were selected to represent seven major scaffolds in GQD. These compounds were labeled in the HPLC fingerprint of GQD (Fig. 2).

### Flavonoid *C*-glycosides (*A*)

*Pueraria* compounds **P2**, **P9** and **P10** were chosen as representatives of this type (Fig. 3). They were abundant in Puerariae Lobatae Radix, and were considered as its characteristic and bioactive constituents^12^. *C*-glycosides could lose the sugar residue to produce corresponding aglycones, which is difficult for chemical hydrolysis. As shown in Fig. 4, **P2**, **P9** and **P10** could be metabolized into 3′-methoxydaidzein, daidzein and 3′-hydroxydaidzein, respectively. This reaction was also observed for isoflavone *C*-diglycoside (**P6**) and flavone *C*-diglycoside (**S4** from Scutellariae Radix). This *C*-glycosidic bond cleavage reaction was firstly reported by Prasain *et al*. and was attributed to microbial metabolism in the intestine, albeit the catalyzing microbial strain is still unknown^22^. **S4** contains both 6-*C*-arabinoside and 8-*C*-glucoside. Only the latter was de-conjugated *in vivo*. The preference could be oriented either by saccharide type or by substitution position. We considered the latter one more reasonable, since both 6-*C*-glc and 8-*C*-glc had been reported for de-conjugation *in vivo*^23^.

Reactions on the backbone were also observed. When the 3′-carbon was substituted, the 3′-OH (3′-methoxydaidzein) and 3′-OCH_3_ (3′-hydroxydaidzein) isoflavones could be transformed into each other, while the 3′-H isoflavone (daidzein) could be reduced to *S*-equol (**P5**). Phase II conjugation reactions (to form glucuronides and sulfates) were common for *C*-glycosides. However, when the glycosides were hydrolyzed into corresponding aglycones, phase II metabolites occurred only on daidzein (**P9**). We speculate that the 3′-H, 4′-OH substitutions may play an important role^24^. Metabolites identified from the three *Pueraria* compounds were listed in Table 1S. Plasma and feces samples mainly contained phase II metabolites and aglycones, respectively, while urine samples covered most metabolites.

Flavonoid *C*-glycosides produced characteristic fragments in (−)-ESI-MS. The *C*-glycosides cleaved to produce neutral loss of C_4_H_8_O_4_ (120 Da) and C_3_H_6_O_3_ (90 Da). Aglycone fragments *m/z* 295, 311, and 325 were produced for **P9**, **P10** and **P2**, after eliminating 120 Da. The aglycone could be cleaved on C-ring to produce *m/z* 253, 269, and 283, respectively. Phase I metabolites of *Pueraria* compounds underwent minor modification on their aglycones, and produced fragment ions that were close to the parent compounds, e.g. *m/z* 241, 257, and 267. Phase II metabolites mainly undertook neutral losses of glucuronide (NL 176) and sulfate (NL 80)^12^,^19^,^25^. Typical MS/MS fragmentations are shown in Fig. 5. PRE and NL ions were then selected from diagnostic fragments (labeled in red and blue, respectively), as listed in Table 1.

### Flavonoid *O*-glucuronides (*B*)

Occurrence of *O*-glucuronides is less common in plants. Flavonoid glucuronides in GQD are all derived from Scutellariae Radix, where they were considered as characteristic components^13^. Three major ones (**S14**, **S15**, **S16**) were chosen to study their *in vivo* metabolism. They underwent hydrolysis in gut, which allowed the absorption of their prototype and aglycone simultaneously. After absorption, aglycones will rapidly conjugate with endogenous glucuronic acid. Thus the interconversion between glucuronides and their aglycones became the major metabolic pathway of *Scutellaria O*-glucuronides^26^,^27^,^28^. Aside from the interconversion, glucuronide relocation in this study was also a major reaction. For **S15** and **S16**, isomers of the parent drug were detected, and their aglycones remained unchanged, as confirmed by *β*-glucuronidase hydrolysis. Hence, we proposed that the glucuronide group was hydrolyzed during absorption, and the *in vivo* conjugation occurred at a different position. We speculate that the glucuronide group was relocated from C-7 to C-5 for **S15** (5-OH, 8-OCH_3_), and from C-7 to C-6 for **S16** (5-OH, 6-OH), according to previous report^29^. Several glucuronide conjugates have now been proved for bioactivities, including wogonoside (**S15**) baicalin (**S16**), and scutellarin (**S13**)^27^,^28^. Changes in substitution positions may alter the bioactivity of the parent drug. Although glucuronidation occurred readily, sulfate products were rarely detected. Metabolic pathways of these three compounds are shown in Fig. 6.

Other metabolic reactions for glucuronides included methylation/demethylation at C-6 and C-8, and glycosidation on the backbone^26^. For example, **S16** could produce glycoside SS21 (*m/z* 431→269, 197, identified as baicalein-*O*-glc) and glucuronide-conjugated glycoside SS10 (*m/z* 607→431→269, identified as baicalein 7-*O*-glc-*O*-gluA). These metabolites were detected in urine and plasma, and the unconjugated form of SS10 was confirmed as baicalein-7-*O*-glucoside (Fig. 6). Glycosidation is a rare *in vivo* reaction, and was reported to be catalyzed by human liver microsomes^30^. Metabolites derived from the three *Scutellaria* compounds were listed in Table 2S.

Flavonoid *O*-glucuronides yielded similar MS fragments (NL 176) as their metabolites Besides, flavones, flavanonols and flavonols showed various fragmentation pathways, which could be explained according to previous studies^14^,^19^,^31^. Characteristic fragments were summarized for *Scutellaria* compounds (Fig. 7). Although MS behaviors are similar for natural glucuronides and their metabolites, natural products haveve unique substitution at the C-7 position, while *in vivo* metabolites generally contain C-5 gluA substitution. These isomers could be separated by HPLC. Based on the MS fragmentations, NL and PRE scan channels for metabolites screening were listed in Table 1.

### Benzylisoquinoline alkaloids (*C*)

**Figure 1:**
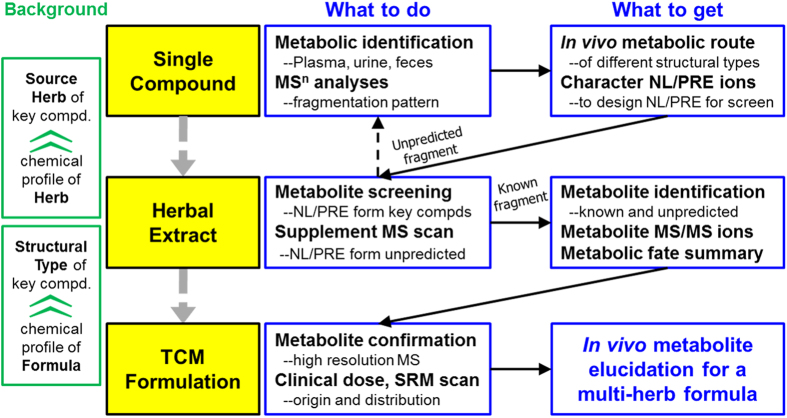
Knowledge-transmitting strategy of the study.

**Figure 2:**
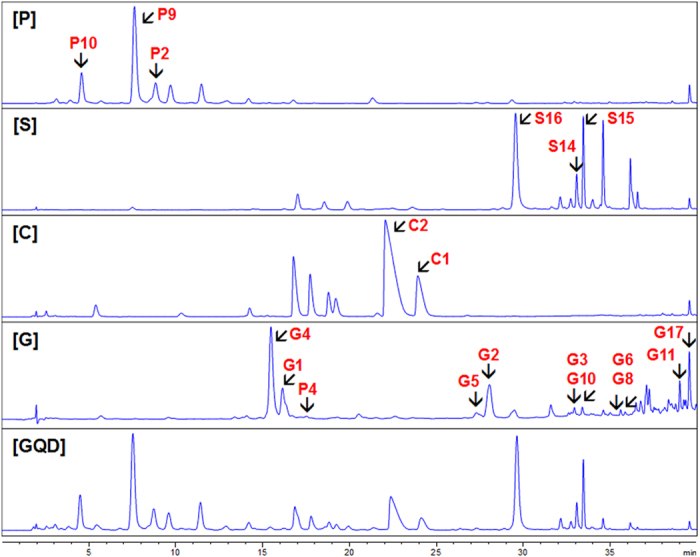
HPLC fingerprints of GQD and its component herbs, showing the single compounds selected for metabolic studies. P, Puerariae Lobatae Radix; S, Scutellariae Radix; C, Coptidis Rhizoma; G, Glycyrrhizae Radix et Rhizoma Praeparata cum Melle. UV wavelength of P, S, C, G and GQD were 254 nm, 270 nm, 270 nm, 300 nm, and 270 nm, respectively. Black arrows show representative compounds.

More than 10% (w/w) of Coptidis Rhizoma crude drug was benzylisoquinoline alkaloids^14^. Their distribution and metabolism had been reported in several different organisms^32^,^33^,^34^,^35^,^36^. We chose palmatine (**C1**) and berberine (**C2**) for metabolic studies. **C2** was found poorly exposed in plasma. Due to lack of free hydroxyl group, **C2** was destined to be deduced (C-2, C-3) or demethylated (C-9, C-10) to form free hydroxyl groups, which then underwent phase II metabolism^36^. **C2** was mainly eliminated from urine, and the metabolic pathway is shown in Fig. 1S. Among multiple metabolites, CS13 (C-10 demethylation), CS15 (C-9 demethylation) and CS8 (glucuronide of CS15) were the major products^33^. **C1** exhibited higher plasma exposure than **C2**. The methoxyl groups at C-2, 3, 9 and 10 underwent multiple demethylation, followed by glucuronide conjugation^34^. Metabolites identification for Coptidis alkaloids were listed in Table 3S. They produced one or multiple common small fragments like H_2_O, CH_3_, and C_2_H_6_N, respectively, and were monitored in the (+)-ESI mode (Fig. 8). Their NL and PRE scans were also preformed in the (+)-ESI mode.

### Free Flavonoids (*D*)

Free flavonoids have complex group members which could be further divided into isoflavones (******D1******), flavones (******D2******), flavanones (******D3******), and chalcones (***D4***). Their metabolism had been studied in our previous report on licorice^20^, represented by compounds **G18**, **G3**, **G10** and **G8**, respectively. Sub-groups ***D1*** to ***D4*** cover major aglycone structures in GQD, and their metabolic pathways could also be supplemented by corresponding glycosides **P2**, **P9**, **P10** (aglycones **P18**, **P3**, **P17** belong to ***D1***), **S14**, **S15**, **S16** (aglycones **S3**, **S2**, **S10** belong to ***D2***), **G4**, **G1** (aglycone **G10** belongs to ***D3***), and **G5**, **G2** (aglycone **G8** belongs to ***D4***).

In brief, metabolism of free flavonoids varies according to the substitution on the backbones. For example, daidzein (**P3**, the aglycone of **P9**) from *Pueraria* could undertake different phase I reactions, including methylation, reduction, and C-ring cleavage. The metabolites were identified as 3′-methoxydaidzein (**P18**), formononetin (**P20**), and *O*-desmethylangolensin (**P7**), respectively, which could be further metabolized into phase II conjugates (Fig. 4). Flavones from *Scutellaria* were mainly involved in (de)methylation and phase II metabolism. Therefore, besides neutral loss of common phase II metabolites (glucuronide and sulfate), common fragments in the flavonoid backbones (CO_2_, CO, CH_3_, and H_2_O) were set for further metabolites screening.

### Flavonoid *O*-glycosides (*E*), coumarins (*F*), and triterpenoid saponins (*G*)

**Figure 3:**
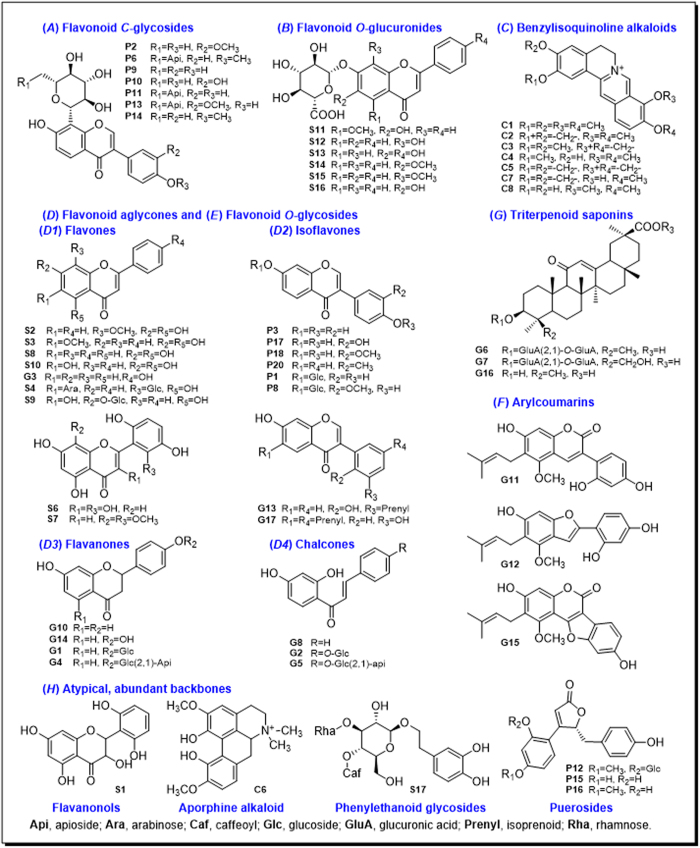
Compounds isolated from Gegen-Qinlian-Decoction (GQD).

Flavonoids and saponins were major constituents of licorice. Our previous studies elucidated metabolism of 11 single compounds^20^,^21^. These compounds were labeled in Fig. 3. Briefly, major metabolic reactions for types *****E****, ***F****, and ****G**** were de-glycosidation, phase II conjugation, and de-glucuronidation, respectively. For example, **P1** (daidzin, daidzein 7-*O*-glucoside) was rapidly hydrolyzed into daidzein (**P3**) following absorption^24^,^37^. Flavonoid *O*-glycosides and triterpene saponins underwent hydrolysis after oral administration to produce corresponding aglycones. Flavonoids were eliminated more rapidly than saponins^21^. Similar to *Scutellaria* glucuronides, flavonoid *O*-glycosides could produce neutral loss fragment of glycoside (162 Da), and precursor ions of [A-H]^−^ (A = aglycone) in the negative ion mode. Notably, saponins behaved oppositely in CID, where the aglycone fragment (e.g. 470 Da for **G6**) was neutral while the saccharide chain (*m/z* 351) was charged. Coumarins could uniquely lose the CO_2_ group (44 Da). These fragments were considered as characteristic NL/PRE ions, and were used in metabolites screening of GQD.

### Atypical and abundant backbones (*H*)

Besides major structural types (***A***) to (***G***), atypical structures were also found in GQD, according to previous chemical studies^19^. They were also taken into consideration in our study, using the metabolite screening method. Representative scaffolds include flavanonol (**S1**, 3,5,7,2′,6′-pentahydroxyflavanone), phenylethanoid glycosides (**S17**, acteoside), puerosides (**P12**, sophoraside A), aporphine alkaloids (**C6**, magnoflorine), and flavonols (**S7**, viscidulin III). Metabolites identification of **S1**, **C6**, **S17**, **P12** were depicted in Fig. 9. In general, metabolites through a common pathway were discovered by NL scans (80, 176 and 162 Da). These metabolites were screened to rule out false positive by a complementary EIC scan (e.g. EIC 477, 479, and EIC 623, respectively), and were identified by corresponding PRE scan (*m/z* 311, *m/z* 301, 303 and *m/z* 299, respectively). For the aporphine alkaloid **C6**, common NL scan of 176 Da (*m/z* 518) obtained signals at around 14 min. However, the other common NL (18 Da, H_2_O) of alkaloid did not respond to this signal compared with benzylisoquinoline alkaloids, suggesting minor differences in the structure. The metabolite was finally identified as magnoflorine glucuronide. The parent compound was confirmed by enzyme hydrolysis and authentic reference compounds. Metabolite identification of **S7** using the similar method is described in the following section. For the eight atypical scaffolds, major NL/PRE scans, major metabolic reactions and metabolite distributions were summarized as in Table 1.

### Metabolism of herbal extracts

Based on the fragmentation pathways summarized from representative single compounds, NL and PRE scans were established to search for conjugation groups and aglycone groups, respectively^38^,^39^. These scans were used to confirm identified metabolites and to discover new metabolites as a complementary method. By the scanning of familiar conjugates, unfamiliar aglycones might be exhumed, and *vice versa*. Once an unfamiliar aglycone was found, a new PRE scan would be established to search for its metabolites. Workflow of this step was depicted in Fig. 10. For the component herb Scutellariae Radix, 18 PRE scans were newly established for *Scutellaria* compounds following the above strategy. In total 30 PRE scans and 9 NL scans allowed the identification of 56 metabolites, after an oral administration of Scutellariae Radix extract (Table 4S). Here we take viscidulin III-*O*-sulfate (SE24, SE44) as an example to elucidate the process. Firstly, according to the diagnostic ions for ***A*** to ***H*** (Fig. 7), NL 80 and PRE 431 were used to analyze rats plasma samples. A number of metabolites could be detected by NL 80 (Fig. 10), and their charged fragments (aglycones) could be calculated as [M-H-80]^−^, where [M-H]^−^ was the parent ion detected in neutral loss scan.

Therefore, the charged aglycone of an *m/z* 511 metabolite (RT = 58.4) was calculated as *m/z* 431. The signal was in accordance with the PRE 431 scan, where the peak *m/z* 511 was detected at the same retention time (Fig. 10). The metabolite was identified as baicalein-*O*-glc-*O*-sulfate (SE40) based on the studies of single compounds. At the same time, a pair of *m/z* 425 signals (RT = 43.6, 59.1) was detected by NL 80, suggesting their charged aglycone as *m/z* 345. Therefore, a PRE *m/z* 345 scan was newly established, from which the corresponding signals were found (RT = 43.6 min, 59.1 min). Meanwhile, a group of phase II conjugates derived from *m/z* 345 were detected (Fig. 11). These conjugates could be degraded by *β*-glucuronidase, producing a single aglycone, which was identified as viscidulin III by comparing with a reference standard. According to the correspondence of NL 80 and PRE 345, SE24 and SE44 were identified as viscidulin III-*O*-sulfates. Other metabolites were identified as viscidulin III-*O*-GluA-*O*-Sul (SE10) and viscidulin III-*O*-GluA (SE12, SE16). The 12 supplemented PRE ions are shown in Table 1. The correlation of all NL and PRE scans were visualized in Fig. 11.

Likewise, we established 14 new PRE scans for Puerariae Lobatae Radix and Coptidis Rhizoma (5S, 6S, and 7S). For example, magnoflorine (*m/z* 342) was found by the neutral loss scan of 45 Da. Its diagnostic signals *m/z* 342 and *m/z* 297 were used in successive PRE scans. A signal (RT = 14.0 min) was consistent in PRE 342, PRE 297 and NL176 scans, and was then identified as magnoflorine-*O*-GluA (CE5, confirmed by *β*-glucuronidase hydrolysis). As a result, in total 56, 47 and 31 metabolites were identified from Scutellariae Radix, Puerariae Lobatae Radix, and Coptidis Rhizoma, respectively (Tables 4S, 6S and 7S). Metabolites information of Glycyrrhizae Radix et Rhizoma Praeparata cum Melle were obtained from our previous study.

### Metabolites identification of Gegen-Qinlian Decoction

Firstly, high-resolution mass spectrometry (HRMS) was employed to further confirm the formula of GQD metabolites. HRMS was performed using a qTOF-MS instrument. Mass error of all metabolites were not higher than 5 ppm, showing an agreement with the identified structure (Tables 4S, 6S, 7S). Then, a LC-SRM/MS method was established (Table 2, Table 8S) to detect 131 metabolites after oral administration of GQD (60 g/kg). Notably, when the dosage was reduced to approach clinical use (12.5 g/kg, equivalent to 6.26 g/kg of P, 2.34 g/kg of S and C, 1.56 g/kg of G), 112 metabolites could still be detected in rats plasma and urine samples. Source herb and *in vivo* distribution of these metabolites are shown in Table 9S. Thus far, the stepwise identification of GQD metabolites was achieved.

## Discussion

*In vivo* metabolites of natural products have comparative, even favorable chances to initiate therapeutic effects as the unchanged form. Therefore, we established the new strategy “compound to extract to formulation”, which could be used to systematically identify the metabolites of Chinese medicine formulas. Although a number of reports are available on metabolites identification of Chinese medicine formulas, most of the studies directly characterized the metabolites by LC/MS techniques. Structural characterization of the metabolites may not be solid enough due to limited structural information provided by mass spectrometry. In our work, the metabolites were not only identified by mass spectrometry, but also according to the metabolic rules of different types of compounds. The metabolites of the formula were also compared with those of single component herbs.

**Figure 4:**
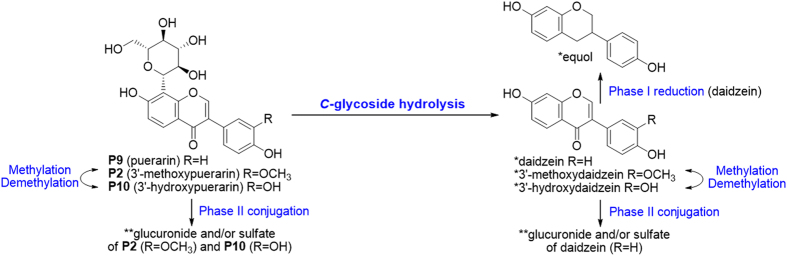
Metabolic pathways of flavonoid *C*-glycosides P2, P9 and P10. Metabolites were identified by authentic reference compounds before (*) or after (**) *β*-glucuronidase hydrolysis.

**Figure 5:**
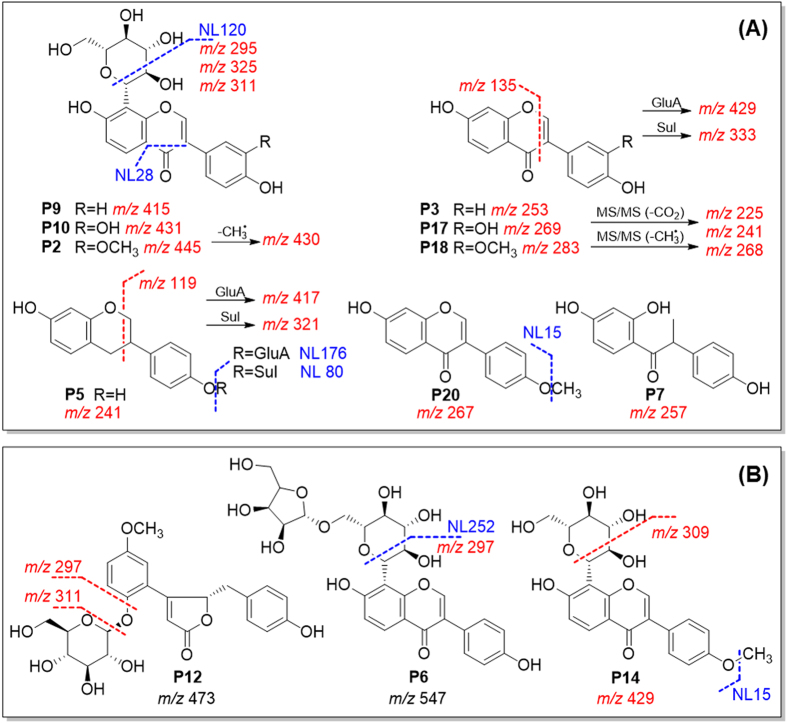
Neutral loss scans (mass value in blue) and precursor ion scans (*m/z* in red) established from predicted (**A**) and unpredicted (**B**) metabolites for Puerariae Lobatae Radix.

**Figure 6:**
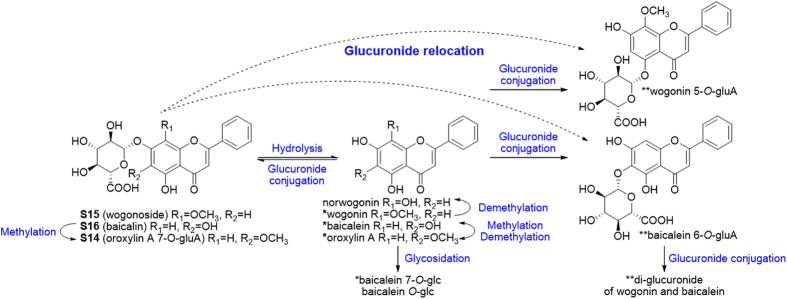
Metabolic pathways of flavonoid *O*-glucuronides S14, S15 and S16. Metabolites were identified by authentic reference compounds before (*) or after (**) *β*-glucuronidase hydrolysis.

**Figure 7:**
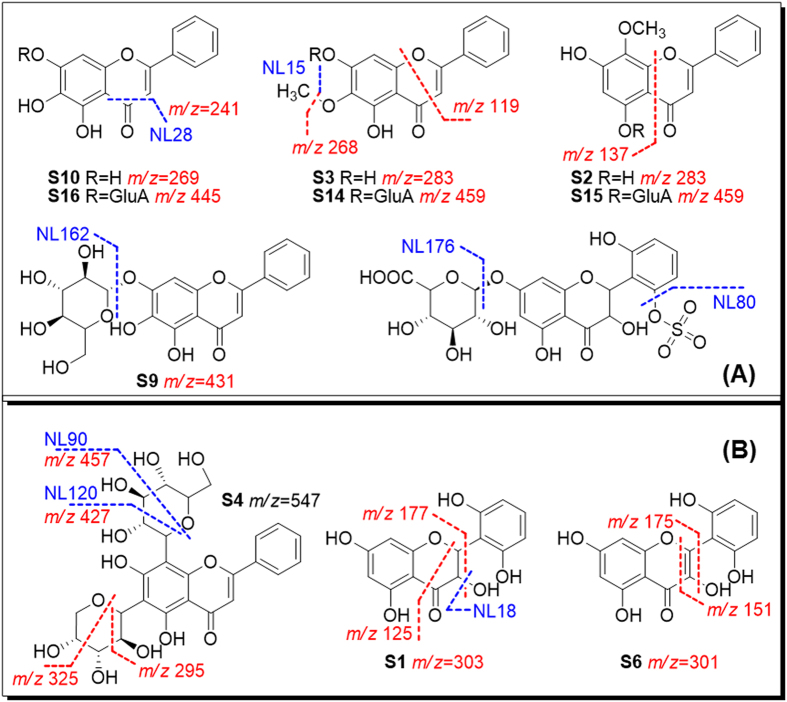
Neutral loss scans (mass value in blue) and precursor ion scans (*m/z* in red) established from predicted (**A**) and unpredicted (**B**) metabolites for Scutellariae Radix.

**Figure 8:**
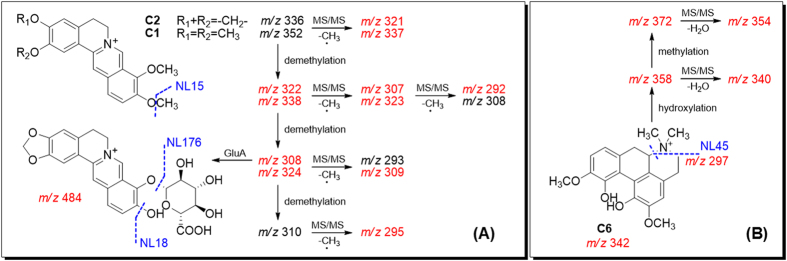
Neutral loss scans (mass value in blue) and precursor ion scans (*m/z* in red) established from predicted (**A**) and unpredicted (**B**) metabolites for Coptidis Rhizoma.

**Figure 9:**
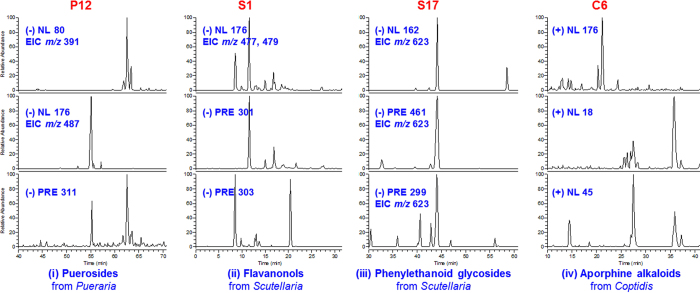
Metabolites identification of P12(i), S1(ii), S17(iii), and C6(iv) using NL and PRE scans.

**Figure 10:**
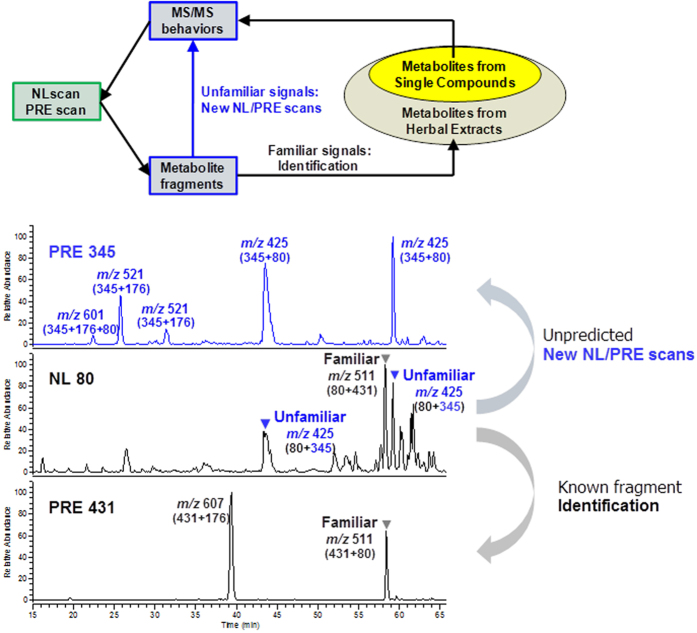
Workflow (top) and an example (bottom) for metabolites screening in herbal extracts.

**Figure 11:**
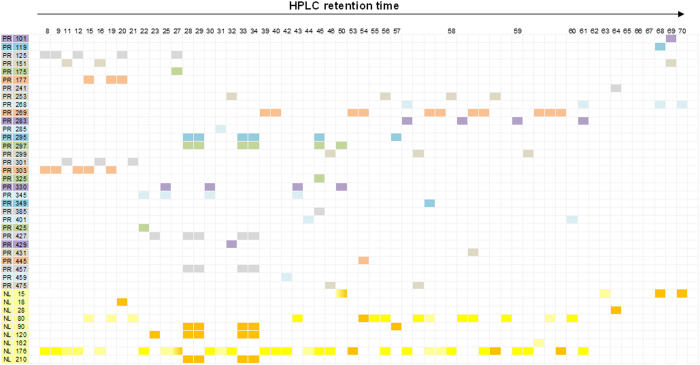
Correlation of all NL and PRE scans for metabolites identification of Scutellariae Radix.

To the specific formulation GQD, we had identified 42 metabolites using LC/MS techniques^19^. We had also established a “Compound to Extract” strategy to discover the metabolites in licorice, one component herb in GQD^20^. However, the complexity of GQD was not fully represented, given that at least 138 compounds could be identified in GQD by 2D-LC/MS. Thus, the metabolic pathways of other representative compounds should be investigated to comprehensively identify the metabolites of GQD. Therefore, we established the knowledge-transmitting approach addressing the complexity of this four-herb formulation. The strategy contains the following steps:

- Background knowledge of the target TCM formulation. We use the information to divide the formula into 8 chemical groups (***A*** to ***G***), and to select representative single compounds.
- Metabolite identification of 19 single compounds. Metabolites (in rats plasma, urine and feces) were identified by the aid of UV spectra, HPLC retention, tandem MS, *β*-glucuronidase hydrolysis, and comparison with reference standards. MS fragmentation pattern were also revealed in this step. We proposed the metabolic routes (as in Figs 4, 6 and 1S) and obtained 57 diagnostic NL/PRE ions (22, 21 and 18 for P, S, and C, respectively, as in Figs 5, 7 and 8).
- Metabolites screening for herbal extracts. NL/PRE scans were used to confirm known fragments and to exhume unpredicted metabolites (Fig. 9). The process (Fig. 10) established 8, 18 and 8 NL/PRE channels for P, S, and C, respectively. Metabolites were identified by matching the screening data in NL and PRE modes. SRM transitions of the metabolites were obtained. The metabolic fate of chemically different compounds were summarized in Table 1.
- Monitoring the metabolites for the four-herb formula. The metabolites were confirmed by HRMS, and were monitored in rats plasma by LC-SRM/MS (Table 2). In total 131 metabolites were detected at a high (60 g/kg) dose, including 46 phytochemicals and 85 newly formed ones. Among them, 112 metabolites could be detected in clinical (12.5 g/kg) dosage.

Different from most metabolite identification reports, the metabolic routes for different scaffolds in GQD were addressed, and the information was transferred from compound to formula with less loss or redundancy. Moreover, parent compounds for the detected metabolites were interpreted. On the other hand, background knowledge for chemical constituents in the formulation was required to use this strategy, which might be less convenient for extracts whose components were unknown.

The strategy allowed systematic discovery of GQD metabolites. In our previous study, 42 metabolites could be identified by LC/MS^n^ for Gegen-Qinlian-Wan, following a high dose of administration (66.6 g/kg). Detectable metabolites could be fewer than 20 using a clinical dose. In the current study, 131 metabolites were identified following the knowledge-transmitting strategy. Among them, 36 were identified by comparison with authentic references, and 81 could be identified after *β*-glucuronidase hydrolysis (45 compared with reference standards). Particularly, *in vivo* exposure of *Pueraria* puerosides and *Scutellaria* pentahydroxyflavanones had yet to be reported, and they were revealed by using our NL/PRE metabolic screening method. In total 34 metabolites were unchanged constituents from GQD, as shown in Table 2. These compounds could be important to the pharmacological effect of GQD.

**Figure 12:**
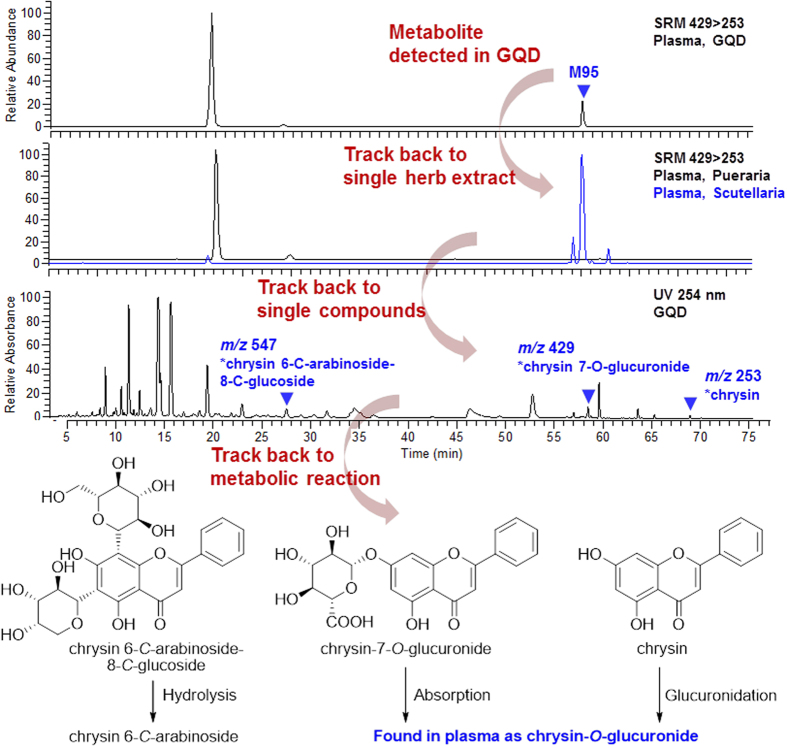
A trace back from metabolite M95 to its parent compounds in GQD.

The “Compound to Extract to Formulation” strategy also provided reliable and traceable metabolites identification. For a metabolite, the strategy could be helpful to trace back to its source herb and source compound. For instance, in the plasma of GQD-treated rats, two metabolites were detected by the SRM pair *m/z* 429 > 253 (Fig. 12). Compared with the component herbs, the metabolites were derived from Puerariae Lobatae Radix (RT = 20.6 min, M29) and Scutellariae Radix (RT = 58.0 min, M95), respectively. In succession, Scutellariae Radix-treated rats plasma were hydrolyzed by *β*-glucuronidase, and the aglycone of M95 was identified as chrysin. Based on the chemistry of Scutellariae Radix^13^, chrysin backbone was involved in three types of constituents, including flavonoid di-*C*-glycosides (represented by **S4**), chrysin 7-*O*-GluA (**S5**), and chrysin (**S8**). Based on the knowledge from single compounds, de-conjugation of the *C*-arabinoside was difficult *in vivo*, thus **S4** was less possible to be the parent compound of M95. Meanwhile, **S5** could be absorbed and exposed in plasma, while chrysin (**S8**) could be easily transformed into its glucuronide-conjugated from. Finally, the metabolic route for M95 is elucidated in Fig. 12. Similarly, the 131 metabolites of GQD could be tracked back to 52 parent phytochemicals. These compounds were listed in Table 2.

## Experimental

### Materials and reagents

HPLC grade acetonitrile and formic acid (J. T. Baker, Phillipsburg, NJ) were used for LC/MS analysis. De-ionized water was purified by a Milli-Q system (Millipore, Bedford, MA). Other solvents were of analytical grade. Daidzin (**P1**), 3′-methoxypuerarin (**P2**), daidzein (**P3**), ononin (**P4**), formononetin 8-*C*-apiofuranosyl(1,6)glucoside (P6), 3′-methoxydaidzin (**P8**), puerarin (**P9**), 3′-hydroxypuerarin (**P10**), mirificin (**P11**), (4*S*)-puerol B-2′′-*O*-glucopyranoside (**P12**), 3′-methoxymirificin (**P13**), 4′-methoxypuerarin (**P14**), 3′-hydroxydaidzein (**P17**), 3′-methoxydaidzein (P18), dihydrodaidzein (**P19**), and formononetin (**P20**) were isolated from Puerariae Lobatae Radix. (2*R*,3*R*)-3,5,7,2′,6′-Pentahydroxyflavanone (**S1**), wogonin (**S2**), oroxylin A (**S3**), chrysin 6-*C*-arabinoside-8-*C*-glucoside (**S4**), chrysin 7-*O*-glucuronide (**S5**), 3,5,7,2′,6′-pentahydroxyflavonol (**S6**), chrysin (**S8**), baicalein 7-*O*-glucoside (**S9**), baicalein (**S10**), lateriflorein 7-*O*-glucuronide (**S11**), norwogonin 7-*O*-glucuronide (**S12**), oroxylin A 7-*O*-glucuronide (**S14**), wogonoside (**S15**), baicalin (**S16**), and acteoside (**S17**) were isolated from Scutellariae Radix. Liquiritin (**G1**), isoliquiritin (**G2**), 7,4-dihydroxyflavone (**G3**), liquiritin apioside (**G4**), isoliquiritin apioside (**G5**), glycyrrhizic acid (**G6**), licorice saponin G2 (**G7**), isoliquiritigenin (**G8**), liquiritigenin (**G10**), glycycoumarin (**G11**), licocoumarone (**G12**), licoisoflavone A (**G13**), glycyrol (**G15**), and isoangustone A (**G17**) were isolated from Glycyrrhizae Radix. All the above compounds were purified by the authors, and the structures were identified by UV, MS, and NMR spectroscopic analyses. Palmatine (**C1**), berberine (**C2**), epiberberine (**C3**), jatrorrhizine (**C4**), coptisine (**C5**), magnoflorine (**C6**), and scutellarin (**S13**) were purchased from Mansite Bio-Technology Co., Ltd. (Chengdu, China). Demethyleneberberine (**C8**) was from Feiyu Fine Chemical (Jiangsu, China). Berberrubine (**C7**), naringenin (**G14**) and glycyrrhetinic acid (**G16**) were purchased from Zelang Co. Ltd. (Nanjing, China). Viscidulin III (**S7**) was purchased from BioBioPha Co. Ltd (Yunnan, China). Puerol A (**P15**) and puerol B (**P16**) were obtained by hydrolysis of (4*S*)-puerol A 2′′-*O*-glucopyranoside and (4*S*)-puerol B-2′′-*O*-glucopyranoside (**P12**) (isolated from P), respectively. Davidigenin (**G9**) was synthesized as previously reported^21^. *O*-Desmethylangolensin (**P7**) was kindly donated by Professor Xiu-ling Wang at Hebei Agricultural University. *S*-Equol (**P5**) and *β*-glucuronidase (HP-2 type) were purchased from Sigma-Aldrich (St. Louis, MO). All the above reference compounds showed purities of >98% by HPLC/UV analysis. Their structures are given in Fig. 3.

Puerariae Lobatae Radix (P), Scutellariae Radix (S), Coptidis Rhizoma (C), and Glycyrrhizae Radix et Rhizoma Praeparata cum Melle (G) were purchased from TianHeng pharmacy (Beijing, China). They were identified as dried roots of *Pueraria lobata* (Willd.) Ohwi, dried roots of *Scutellaria baicalensis* Georgi, dried rhizomes of *Coptis chinensis* Franch., and dried roots and rhizomes of *Glycyrrhiza uralensis* Fisch. (stir-baked with honey), respectively, according to the Chinese Pharmacopeia (2015 edition)^10^. GQD decoction was prepared according to its original record in *Shang-Han-Lun.* The four crude drug materials (slices) were separately decocted in 10-fold volume of water for three times (1.5 h, 1.5 h, 0.5 h) to obtain the extracts. GQD was prepared by extracting the four component herbs (P:S:C:G = 8:3:3:2) using the same method as described above for the single herbs, after a pre-extraction of Puerariae Lobatae Radix for 0.5 h. For each extract, the decoctions were filtered to remove the herbal residue, combined, and concentrated in vacuum at 50 °C. Final concentrations of the extracts were 0.8 g/mL for Puerariae Lobatae Radix, 1.5 g/mL for Scutellariae Radix, 0.75 g/mL for Coptidis Rhizoma, and 1.6 g/mL for GQD (crude drug per g/mL).

### Animals

Male Sprague-Dawley rats (8–10 weeks, 180–250 g) were provided by Experimental Animal Center of Peking University Health Science Center (Beijing, China). The rats were kept in a controlled environment at 25 °C, 60 ± 5% humidity and a 12-h dark-light cycle for 10 days, with free access to water and normal diet. Animals treated with *Pueraria* compounds, Puerariae Lobatae Radix, and GQD were fed with soy-free custom diet (Ke’ao Xieli Co., Beijing, China) to avoid disturbance of isoflavones in soybean^23^. All animals were fasted for 12 h before treatment. The animal facilities and protocols were approved by the Animal Care and Use Committee of Peking University Health Science Center. All procedures were in accordance with Guide for the Care and Use of Laboratory Animals (National Institutes of Health).

All single compounds were dissolved or suspended in 0.03% carboxymethylcellulose sodium salt solution, and were then orally administered to rats (40 mg/kg). Herbal extracts were administrated separately at two doses, 1.2 and 16 g/kg for Puerariae Lobatae Radix, 0.6 and 6 g/kg for Scutellariae Radix, 0.5 and 6 g/kg for Coptidis Rhizoma, and 12.5 and 60 g/kg for GQD (crude drug per g/kg of body weight), respectively. Dosage ratio among component drugs followed their composition in GQD, and the high dosages are roughly 10 folds of low ones (13, 10 and 12 folds for P, S, C), respectively. Medication groups received 4.0, 0.8, 1.6 and 7.5 mL decoction of *Pueraria, Scutellaria, Coptidis*, and GQD, respectively. The control group received 2 mL of normal saline.

Retro-orbital blood (400 μL) were collected into heparinized tubes at 0.25, 0.5, 1.5, 4, 6, 8 and 12 h after administration (*n* = 2). Blood samples were immediately centrifuged at 6000 rpm (4 °C) for 20 min. The supernatant was separated and combined as pooled plasma samples. Urine and feces samples were collected over 0–12 h and 12–24 h periods (*n* = 2), and then combined. All samples were stored at −20 °C until analysis.

### Sample preparation

*Plasma* (pooled) – 4 mL of plasma was mixed with 12 mL of methanol. The mixture was vortexed at 2200 rpm for 5 min, and then centrifuged (9000 rpm, 4 °C) for 10 min. The supernatant was separated and dried under a gentle flow of nitrogen at 40 °C. *Urine* (pooled) – 4 mL of urine was centrifuged (9000 rpm, 4 °C, 5 min) and loaded onto an Oasis^®^ HLB SPE column (6 cc, pre-eluted with 6 mL of methanol and 6 mL of de-ionized water, successively). The samples were eluted with 3 mL of de-ionized water, 3 mL of 5% methanol, and 5 mL of methanol in succession. The methanol fraction was collected and dried under a gentle flow of nitrogen at 40 °C. *Feces* (pooled) – 0.5 g of dried sample was extracted with 10 mL of methanol in an ultrasonic bath for 30 min, and centrifuged (9000 rpm, 4 °C) for 10 min. The supernatant was collected and dried under a gentle flow of nitrogen at 40 °C. Residues of plasma, urine and feces were stored at −20 °C until use. Samples were reconstituted and diluted differently according to analytical method, which was described as follows. All samples were filtered through a 0.22-μm membrane. The combination of different pre-treatment, separation and detection methods was summarized in Table 3.

- Samples from single-compound administrated animals were reconstituted in 500 μL of methanol before analysis.
- Samples from herbal extract administrated animals were reconstituted in 500 μL of methanol, and then diluted for 5, 5, and 10 folds (for plasma, urine and feces samples).
- Samples from single-compound administrated animals were reconstituted in 1000 μL of methanol before analysis.

### Enzyme hydrolysis

The structures of glucuronides were confirmed by enzyme hydrolysis. The plasma or urine sample (50 μL) described under “Sample preparation” section was dried under nitrogen flow (40 °C) and mixed with 200 μL of β-glucuronidase solution (containing 19.86 U, in sodium acetate buffer, pH 5.5). The mixture was vortexed at 2200 rpm for 5 min, incubated at 37 °C for 2 h, mixed with 800 μL of methanol, and prepared using the same method as plasma samples as described in “Sample preparation” section. The residue was reconstituted in 300 μL of methanol before analysis.

### HPLC analysis

A Finnigan Surveyor LC instrument was employed (ThermoFisher, CA, USA). The mobile phase consisted of acetonitrile (A) and water containing 0.1% formic acid (B).

- An Atlantis T3 column (3 μm, ID 2.1 × 150 mm) equipped with an XTerra MS C_18_ guard column (5 μm, ID 3.9 × 20 mm) (Waters, MA, USA) was used. The gradient elution program was used as follows: 0–8 min, 5–25% A; 8–12 min, 25% A; 12–15 min, 25–40% A; 15–23 min, 40–80% A; 23–25 min, 80–95% A; 25–27 min, 95% A. The flow rate was 200 μL/min. The HPLC effluent was introduced into the mass spectrometer without splitting. The column temperature was 30 °C. An aliquot of 5 μL was injected for analysis.
- An Agilent Eclipse XDB C_18_ column (5 μm, 4.6 × 250 mm) equipped with a Zorbax SB C_18_ guard column (5 μm, 4.6 × 12.5 mm) was used. The gradient elution program was as follows: 0–10 min, 5–12% A; 10–40 min, 12–19% A; 40–50 min, 19–20% A; 50–70 min, 20–55% A; 70–75 min, 55–90% A; 75–80 min, 90–100% A. The flow rate was 1000 μL/min. The column temperature was 30 °C. The post-column splitting ratio was 3:1. An aliquot of 10 μL was injected.

### Tandem mass spectrometry

A Finnigan LCQ Advantage ion trap mass spectrometer equipped with an ESI interface (Thermo Finnigan, San Jose, CA, USA) was used. Collision gas, high purity helium (He); nebulizing gas, high purity nitrogen (N_2_). Source-dependent parameters were as follows: sheath gas (N_2_), 50 arb; auxiliary gas (N_2_), 15 arb; spray voltage, 4.5 kV; capillary temperature, 330 °C; capillary voltage, 3 V/−4 V (positive/negative mode); tube lens offset voltage, 30 V/−60 V (positive/negative mode). MS full scan range, *m/z* 120–1500; Collision energy for CID, 35%; Source-fragmentation voltage, 0 V/25 V (positive/negative mode).

### Neutral loss scan, precursor ion scan, and SRM scan

A Finnigan TSQ Quantum triple quadrupole mass spectrometer was connected to the HPLC *via* ESI interface (ThermoFisher, CA, USA). The mass spectrometer was operated in the negative and positive ion modes. High purity nitrogen was used as the sheath and auxiliary gas; high purity argon was used as the collision gas (1.5 mTorr). Q1 and Q3 quadrupoles were set at unit resolution. Tune parameters and NL/PRE ions were described in Table 5S.

### UHPLC-DAD-qTOF-MS analysis

An Agilent series 1290 UHPLC instrument (Agilent, Waldbronn, Germany) was coupled with a 6538 qTOF mass spectrometer (Agilent Technologies, Santa Clara, CA) *via* an ESI interface. The UHPLC instrument was equipped with a binary pump, a diode-array detector, an autosampler, and a column compartment. Samples were separated on a Zorbax Eclipse Plus C_18_ column (2.1 × 100 cm, 1.8 μm). The mobile phase consisted of acetonitrile (A) and water containing 0.1% (*v/v*) formic acid (B). A gradient program was used as follows: 0–10 min, 10–30% A; 10–15 min, 30–50% A; 15–20 min, 50–80% A; 20–22 min, 80–95% A; 22–24 min, 95% A; 24–30 min, 10% A. Flow rate, 300 μL/min; column temperature, 40 °C; injection volume, 2 μL. High-purity nitrogen (N_2_) was used as drying gas (10 mL/min) and nebulizing gas (45 psig), and ultra-high purity helium (He) was used as the collision gas. Both negative and positive ion polarity modes were used for compounds ionization. Gas temperature was 350 °C. Other parameters were as follows: capillary voltage, 4000 V; fragmentor voltage, 130 V; skimmer voltage, 65 V; octopole 1 rf voltage, 750 V; data acquisition, 2 spectra/s. Mass spectra were recorded in the range of *m/z* 150–1000. MS^n^ (n = 2–4) was triggered by a data-dependent threshold. Data were analyzed with MassHunter software (Agilent Technologies).

## Conclusions

A knowledge-transmitting approach was established to elucidate *in vivo* metabolites of Gegen-Qinlian Decoction. The metabolites of GQD was revealed stepwise, from single compounds to component herb, and then to the formulation. The study improved previous ones in the following points: (i) 85 newly formed metabolites were identified and monitored unbiasly; (ii) metabolic fate of different structural types (classified as 8 groups) were revealed globally; and (iii) metabolites could be tracked back to 52 parent phytochemicals. The knowledge-transmitting strategy helped us to analyse *in vivo* metabolites systematically, toward a very complex TCM formulation. Knowledge of these metabolites will be valuable for elucidating the mechanism of action of Gegen-Qinlian Decoction.

## Additional Information

**How to cite this article**: Qiao, X. *et al*. Compound to Extract to Formulation: a knowledge-transmitting approach for metabolites identification of Gegen-Qinlian Decoction, a traditional Chinese medicine formula. *Sci. Rep.* **6**, 39534; doi: 10.1038/srep39534 (2016).

**Publisher's note:** Springer Nature remains neutral with regard to jurisdictional claims in published maps and institutional affiliations.

## Supplementary Material

Supporting Information

## Figures and Tables

**Table 1:** Eight types of major chemical constituents in GQD. The representative compounds, original herb, MS behavior in NL and PRE scans, major *in vivo* metabolic reactions, and distribution.

	Structural type	Compound	Herb^a^	Neutral loss scan^b^ (*m/z*)	Precursor ion scan (*m/z*)	Supplementary precursor ion scan (*m/z*)	Metabolic reaction^c^	Distribution^d^ (P/U/F)
***A***	**Flavonoid** ***C*****-glycoside**	P2, P9, P10	P, S	15, 28, 120, 176, 252^#^	119, 135, 225, 241, 253, 257, 267, 283, 295, 311, 321, 325, 333, 415, 417, 430, 431, 445	149, 268, 269, 297, 309, 429, 461	**H/S/G deM/M (C3′) RC (C-ring)**	P/MP/M_I_P
***B***	**Flavonoid** ***O*****-glucuronide**	S14, S15, S17	S	15, 18, 28, 80, 90, 120, 162, 176, 210	119, 241, 268, 269, 283, 295, 325, 385, 427, 431, 457, 459	101, 125, 151, 175, 177, 253, 285, 299, 301, 303, 330, 345, 349, 401, 425, 429, 445, 475	**H/G/Glc deM/M (C6,7) C-ring stable**	MP/MP/M_A_P
***C***	**Benzylisoquinoline alkaloid**	C1, C2	C	15, 18, 45^#^, 176 (+)	295, 307, 308, 309, 321, 322, 323, 324, 337, 338, 484 (+)	292, 297, 340, 342, 354, 358, 372 (+)	**deM/M/OH G**	T/M/T
*D*	**Flavonoid aglycone**			Supplementary		NL/PRE feature		
	***D1*** Isoflavone	G17	G, P	P17, P18, P3		NL CO_2_, CO, CH_3_,H_2_O, G, S, etc.Common PRE not available.	**deM/M, RC, S/G**	MP/MP/P
	***D2*** Flavone	G3	G, S	S2, S3, S10			**deM/M, G**	T/M/P or T
	***D3*** Flavanone	G10	G	G4, G1			**RC, R**_**H**_**, S/G**	MP/M/M_I_P
	***D4*** Chalcone	G8	G	G5, G2			**RC, R**_**H**_**, S/G**	MP/M/M_I_P
***E***	**Flavonoid** ***O*****-glycoside**			Supplementary		NL/PRE feature		
	mono-*O-*	G1, G2	G, P	P1, P4		NL/PRE: G/A	**H/S/G, RC**	MP/MP/M_A_P
	di-*O-*	G4, G5	G, P	P6		NL/PRE: G/A	**H/S/G, RC**	MP/MP/M_A_P
***F***	**Coumarin**	G11	G			NL G, S, CO_2_	**OH/O**_**deH**_**, S/G**	MP/MP/P
***G***	**Saponin**	G6	G			NL/PRE: A/G	**H, G, OH**	MP/MP/A
***H***	**Atypical, abundant backbones**			Initiate	Confirm	New scan		
	pueroside	P12	P	NL 80, 176	EIC/PROD 391, 487	PRE 311	**Unstable, H/S/G**	M/M/T
	flavanonol	S1	S	NL 176	EIC/PROD 477, 479	PRE 301, 303	**O**_**deH**_**, S/G**	M_II_/MP/P
	phenylethanoid glycoside	S17	S	NL 162	EIC/PROD 623	PRE 461	**Unstable**	T/M/T
	aporphine alkaloid	C6	C	NL 176, 18 (+)	EIC/PROD 342 (+)	NL 45 (+)	**deM/M/OH, G**	T/M/T
	flavonol	S7	S	NL 80	EIC/PROD 345	PRE 431	**R**_**H**_**, S/G**	M_II_/M/P

^a^Origin: P, Puerariae Lobatae Radix; S, Scutellariae Radix; C, Coptidis Rhizoma; G, Glycyrrhizae Radix et Rhizoma Praeparata cum Melle.

^b^MS scan mode: NL, neutral loss scan; PRE, precursor ion scan; PROD, product ion scan; EIC, extracted ion current; (+) determined in (+) ESI mode; data were obtained in (−) ESI mode unless otherwise stated; Scans marked with # were newly established from MS screening process.

^c^Metabolic reaction: G, glucuronidation; H, hydrolysis; M, methylation; deM, demethylation; OH, oxidation (hydroxylation); OdeH, oxidation (dehydrogenation); RH, reduction (hydrogenation); RC, ring cleavage; S, sulfation.

^d^Distribution, P, plasma; U, urine; F, Feces. Analyte type: M, metabolite; MI, phase I metabolite; MII, phase II metabolite; MA, metabolite formed by hydrolysis, namly aglycones; P, prototype (unchanged form) T, trace or low abundance.

**Table 2:** Metabolites of Gegen-Qinlian Decoction identified in this study. Their retention time, SRM transitions, source herb, and distribution in biosamples.

No.	*t*_R_ (min)	SRM^a^	Identification^b^	Plasma^c^	Urine	Feces	Herb^d^	Reaction^e^	ID^f^	Prodrug^g^
M1	8.5	479/125	pentahydroxyflavanone *O*-GluA	/	HL	/	S	II	Δ*	S1
M2	8.8	534/358	monohydroxymagnoflorine *O*-GluA	/	HL	/	C	I, II	Δ	C6
M3	9.4	479/125	pentahydroxyflavanone *O*-GluA	/	HL	/	S	II	Δ*	S1
M4	9.6	534/358	monohydroxymagnoflorine *O*-GluA	/	HL	/	C	I, II	Δ	C6
M5	11.5	477/125	pentahydroxyflavonol *O*-GluA	/	H	/	S	II	Δ*	S6
M6	11.7	591/415	puerarin *O*-GluA	HL	HL	/	P	II	Δ*	P9
M7	11.9	607/431	3′-hydroxypuerarin *O*-GluA	HL	HL	/	P	II	Δ*	P10
M8	12.5	431/311	3′-hydroxypuerarin	/	HL	H	P	NA	*	P10
M9	12.8	548/372	3′-methoxypuerarin *O*-GluA	/	HL	/	C	II	Δ	P2
M10	12.8	621/445	3′-methoxypuerarin *O*-GluA	HL	HL	/	P	II	Δ*	P2
M11	12.9	479/125	pentahydroxyflavanone *O*-GluA	/	HL	/	S	II	Δ*	S1
M12	13.2	534/358	monohydroxymagnoflorine *O*-GluA	/	HL	/	C	II	Δ	C6
M13	13.8	495/415	puerarin *O*-Sul	HL	HL	/	P	II	Δ*	P9
M14	14.0	518/342	3′-methoxypuerarin *O*-Sul	HL	HL	/	C	II	Δ*	P2
M15	14.0	525/445	magnoflorine *O*-GluA	/	HL	/	P	II	Δ*	C6
M16	14.1	593/417	3′-hydroxypuerarin *O*-Sul	/	HL	/	G	II	Δ*	P10
M17	14.1	511/431	liquiritin 7-*O*-GluA	HL	HL	/	P	II	Δ*	G1
M18	14.6	484/308	didemethylepiberberine *O*-GluA	/	HL	/	C	II	Δ	C3
M19	15.5	342/297	magnoflorine	HL	HL	/	C	NA	*	C6
M20	15.6	415/295	puerarin	HL	HL	H	P	NA	*	P9
M21	15.8	660/308	didemethylberberine di-*O*-GluA	HL	HL	/	C	II	Δ	C2
M22	16.6	509/333	daidzein *O*-Sul-*O*-GluA	HL	HL	/	P	II	Δ*	P3
M23	16.9	547/295	mirificin	HL	HL	H	P	NA	*	P11
M24	17.0	445/325	3′-methoxypuerarin	HL	HL	H	P	NA	*	P2
M25	17.1	525/325	methoxypuerarin *O*-Sul	HL	/	/	P	I, II	Δ	P9
M26	17.7	577/325	3′-methoxymirificin	HL	HL	/	P	NA	*	P13
M27	18.0	548/372	methoxymagnoflorine *O*-GluA	/	HL	/	C	II	Δ	C6
M28	19.5	445/325	methoxypuerarin	HL	HL	/	P	I		P9
M29	20.6	429/253	daidzein *O*-GluA	HL	HL	/	P	II	Δ*	P3
M30	20.9	500/324	demethyleneberberine *O*-GluA	/	HL	/	C	II	Δ*	C8
M31	21.4	498/322	thalifendine 10-*O*-GluA	HL	H	/	C	II	Δ	
M32	21.6	514/338	3-methoxydemethyleneberberine 2-*O*-GluA	HL	HL	/	C	I, II	Δ	C8
M33	22.6	601/425	viscidulin III *O*-GluA-*O*-Sul	HL	HL	/	S	II	Δ*	S7
M34	23.0	577/457	chrysin 6,8-di-*C*-glucoside	H	/	/	S	NA		
M35	24.7	511/335	liquiritigenin *O*-GluA-*O*-Sul	HL	/	/	G	II	Δ*	G10
M36	24.9	498/322	berberrubine 9-*O*-GluA	HL	HL	/	C	II	Δ*	C7
M37	25.8	473/297	puerol A *O*-GluA	HL	HL	/	P	II	Δ*	P15
M38	25.8	521/345	viscidulin III *O*-GluA	/	H	/	S	II	Δ*	S7
M39	27.1	322/307	groenlandicine	HL	HL	/	C	NA		
M40	27.6	324/309	demethyleneberberine	/	H	/	C	NA	*	C8
M41	27.8	549/255	liquiritin apioside	H	H	/	G	NA	*	G4
M42	28.1	417/255	liquiritin	H	HL	/	G	NA	*	G1
M43	28.2	431/175	liquiritigenin *O*-GluA	/	HL	/	G	II	Δ*	G10
M44	28.2	513/337	davidigenin *O*-GluA-*O*-Sul	HL	/	/	G	I, II	Δ*	G9
M45	28.2	547/427	chrysin 6-*C*-Ara-8-*C*-Glc	HL	HL	/	S	NA	*	S4
M46	28.5	605/429	4′-methoxypuerarin 7-*O*-GluA	HL	H	/	P	II	Δ*	P14
M47	29.5	431/175	liquiritigenin *O*-GluA	HL	HL	/	G	II	Δ*	G10
M48	30.8	497/241	equol *O*-Sul-*O*-GluA	HL	/	/	P	I, II	Δ*	P5
M49	31.4	521/345	viscidulin III *O*-GluA	HL	/	/	S	II	Δ*	S7
M50	31.9	497/241	equol *O*-Sul-*O*-GluA	HL	/	/	P	I, II	Δ*	P5
M51	32.0	473/297	puerol A *O*-GluA	HL	HL	/	P	II	Δ*	P15
M52	32.0	445/269	3′-hydroxydaidzein *O*-GluA	HL	HL	/	P	II	Δ*	P17
M53	32.0	605/253	chrysin di-*O*-GluA	HL	HL	/	S	II	Δ*	S8
M54	32.5	547/427	isomer of chrysin 6-*C*-Ara-8-*C*-Glc	HL	HL	/	S	NA		
M55	35.3	429/309	4′-methoxypuerarin	HL	HL	H	P	NA	*	P14
M56	35.8	561/309	formonetin 8-*C*-glu(6-1)-Api	HL	HL	/	P	NA	*	P6
M57	36.8	497/241	equol *O*-Sul-*O*-GluA	HL	/	/	P	I, II	Δ*	P5
M58	36.9	320/292	coptisine	HL	/	H	C	NA	*	C5
M59	37.2	338/323	demethyleneberberine	HL	HL	H	C	I	*	C8
M60	37.2	336/321	epiberberine	HL	HL	H	C	NA	*	C3
M61	37.6	322/307	thalifendine	/	HL	/	C	NA		
M62	38.7	338/323	jatrorrhizine	HL	HL	H	C	NA	*	C4
M63	39.4	607/431	baicalein 7-*O*-Glc-*O*-GluA	HL	HL	/	S	II	Δ*	S9
M64	40.1	621/445	baicalein di-*O*-GluA	HL	HL	/	S	II	Δ*	S16
M65	40.2	459/283	3′-methoxydaidzein *O*-GluA	HL	H	/	P	II	Δ*	P18
M66	41.1	417/241	equol *O*-GluA	HL	HL	/	P	I, II	Δ*	P5
M67	42.3	513/337	davidigenin *O*-GluA-*O*-Sul	HL	/	/	G	I, II	Δ*	G9
M68	43.5	635/459	wogonin di-*O*-GluA	HL	HL	/	S	II	Δ*	S2
M69	43.6	377/297	puerol A *O*-Sul	/	HL	/	P	II	Δ*	P15
M70	44.0	577/401	apigenin-8-*C*-Ara *O*-GluA	HL	HL	/	S	II	Δ	
M71	44.1	333/253	chrysin 6-*C*-Ara *O*-GluA	H	HL	/	P	II	Δ	
M72	44.1	561/385	daidzein *O*-Sul	H	/	/	S	II	Δ*	P3
M73	45.3	363/283	3′-methoxydaidzein *O*-Sul	HL	HL	/	P	II	Δ*	P18
M74	45.8	363/283	3′-methoxydaidzein *O*-Sul	HL	HL	/	P	II	Δ*	P18
M75	49.5	336/321	berberine	HL	HL	H	C	NA	*	C2
M76	50.4	297/119	puerol A	/	HL	/	P	NA	*	P15
M77	50.7	345/315	viscidulin III	/	H	/	S	NA	*	S7
M78	52.1	352/337	palmatine	HL	HL	H	C	NA	*	C1
M79	53.2	443/267	formononetin *O*-GluA	HL	HL	/	P	II	Δ*	P20
M80	53.4	487/311	puerol B *O*-GluA	HL	HL	/	P	II	Δ*	P16
M81	54.2	445/269	baicalin	/	HL	/	S	NA	*	S10
M82	54.6	525/445	norwogonin *O*-Sul	HL	HL	/	S	II	Δ	
M83	55.7	433/257	davidigenin *O*-GluA	/	H	/	G	I, II	Δ*	G9
M84	55.9	255/119	dihydrodaidzein	H	HL	/	P	I	*	P19
M85	56.0	487/311	puerol B *O*-GluA	HL	/	/	P	II	Δ*	P16
M86	56.3	253/225	daidzein	HL	HL	H	P	NA	*	P3
M87	56.3	509/253	chrysin *O*-GluA-*O*-Sul	/	H	/	S	II	Δ*	S8
M88	57.0	433/257	*O*-desmethylangolensin *O*-GluA	HL	HL	/	P	I, II	Δ*	P7
M89	57.0	385/295	chrysin 6-*C*-Ara	/	HL	/	S	NA		
M90	57.1	283/268	3′-methoxydaidzein	HL	HL	/	P	NA	*	P18
M91	57.2	459/283	wogonin 5-*O*-GluA	HL	H	/	S	II	Δ*	S2
M92	57.7	555/475	lateriflorein 7-*O*-GluA-*O*-Sul	HL	/	/	S	II	Δ	S11
M93	57.8	525/269	norwogonin *O*-Sul	/	H	/	S	II	Δ	
M94	57.9	445/269	norwogonin 7-*O*-GluA	/	HL	/	S	NA	*	S12
M95	58.0	429/253	chrysin 7-*O*-GluA	H	/	/	S	NA	*	S5
M96	58.0	539/283	oroxylin A 7-*O*-GluA-*O*-Sul	HL	HL	/	S	II	Δ*	S14
M97	58.4	511/431	baicalein *O*-Glc-*O*-Sul	H	H	/	S	II	Δ	
M98	58.5	337/257	*O*-desmethylangolensin *O*-Sul	HL	HL	/	P	I, II	Δ*	P7
M99	58.7	321/241	equol *O*-Sul	HL	/	/	P	I, II	Δ*	P5
M100	58.8	445/269	norwogonin *O*-GluA	HL	HL	H	S	II	Δ	
M101	58.9	447/271	naringenin *O*-GluA	HL	H	/	G	II	Δ*	G14
M102	58.9	525/269	baicalein *O*-GluA-*O*-Sul	HL	/	/	S	II	Δ*	S16
M103	59.0	425/345	viscidulin III *O*-Sul	H	H, L	/	S	II	Δ*	S7
M104	59.2	459/283	oroxylin A 7-*O*-GluA	HL	H	/	S	NA	*	S14
M105	59.4	475/299	lateriflorein 7-*O*-GluA	HL	HL	/	S	NA	*	S11
M106	59.6	431/269	baicalein *O*-glc	/	HL	/	S	NA	*	S9
M107	59.7	705/529	licoisoflavone A di-*O*-GluA	H	/	/	G	II	Δ*	G13
M108	59.9	445/269	baicalein *O*-GluA	HL	HL	H	S	II	Δ*	S16
M109	60.2	481/401	apigenin-8-*C*-Ara *O*-Sul	HL	HL	/	S	II	Δ	
M110	60.3	459/283	wogonoside	HL	H	/	S	NA	*	S15
M111	61.6	337/257	davidigenin *O*-Sul	/	HL	/	G	I, II	Δ*	G9
M112	61.8	515/339	licocoumarone *O*-GluA	HL	/	/	G	II	Δ*	G12
M113	62.0	391/311	puerol B *O*-Sul	HL	HL	/	P	II	Δ*	P16
M114	62.2	271/151	naringenin	/	HL	/	G	NA	*	G14
M115	63.3	311/119	puerol B	H	HL	H	P	NA	*	P16
M116	63.7	337/257	davidigenin *O*-Sul	/	HL	/	G	II	Δ*	G9
M117	63.9	299/284	lateriflorein	/	H	/	S	NA		
M118	64.2	837/351	licorice-saponin G2	H	/	/	G	NA	*	G7
M119	64.2	269/241	baicalein	H	HL	/	S	NA	*	S16
M120	64.6	257/151	davidigenin	H	HL	/	G	I	*	G9
M121	65.4	257/135	*O*-desmethylangolensin	/	HL	/	P	I	*	P7
M122	65.6	821/351	glycyrrhizic acid	H	/	/	G	NA	*	G6
M123	66.2	267/252	formononetin	H	HL	H	P	NA	*	P20
M124	66.3	269/254	dihydrofomononetin	H	HL	/	G	I		P20
M125	66.9	347/267	formononetin *O*-Sul	HL	HL	/	P	II	Δ*	P20
M126	69.6	283/268	wogonin	HL	HL	H	S	NA	*	S2
M127	69.8	253/151	chrysin	/	H	/	S	NA	*	S8
M128	70.7	367/309	glycycoumarin	H	H	H	G	NA	*	G11
M129	70.7	283/268	oroxylin A	HL	HL	H	S	NA	*	S3
M130	73.5	365/307	glycyrol	HL	HL	H	G	NA	*	G15
M131	77.6	469/425	glycyrrhetinic acid	H	/	/	G	NA	*	G16

^a^SRM, metabolites derived from Coptidis Rhizoma were monitored in (+) ESI. Other SRM transitions were detected in (−) ESI, see Table 8S.

^b^Identification: Api, apioside; Ara, arabicoside; GluA, glucuronide; Glc, glucoside; Sul, sulfate.

^c^Distribution, H, detected in high-dose treated biosamples; L, detected in low-dose treated biosamples; /, not detected.

^d^Component herb to produce corresponding analyte. P, Puerariae Lobatae Radix; S, Scutellariae Radix; C, Coptidis Rhizoma; G, Glycyrrhizae Radix et Rhizoma Praeparata cum Melle.

^e^Metabolic reaction: NA, compound was detected as prototype, I, phase I reaction; II, phase II reaction; I, II, multiple reactions.

^f^ID, *, identified by comparing with reference standards; Δ, confirmed as phase II metabolites by *β*-glucuronidase hydrolysis; Δ*, confirmed as phase II metabolites by *β*-glucuronidase hydrolysis, and the aglycone was identified by comparing with reference standards.

^g^Prodrug, proposed parent molecule of the metabolites.

**Table 3:** Application of different analytical methods for single compounds, herbal extracts and TCM formulation.

	Sample preparation	Liquid chromatography^a^	Mass spectrometry^b^
Single compound	**a** (Section I)	HPLC (Section II) **a** 2.1 × 150 mm, 3.5 μm	MS^n^ (Section III), NL, PRE (Section IV)
Herbal extract	**b** (Section I)	HPLC (Section II) **b** 4.6 × 250 mm, 5 μm	NL, PRE (Section IV)
TCM formulation	**c** (Section I)	HPLC (Section II) **b** 4.6 × 250 mm, 5 μm	SRM (Section IV)
		UHPLC (Section V) 2.1 × 100 mm, 1.8 μm	Full Scan, MS/MS (Section V)

^a^Method applied were described in different sections (section I: Sample preparation; section II: HPLC analysis; section III: Tandem mass spectrometry; section IV: Neutral loss scan, precursor ion scan, and SRM scan; section V: UHPLC-DAD-qTOF-MS analysis).

^b^MS^n^, tandem mass spectrometry; NL, neutral loss scan; PRE, precursor ion scan; SRM, selected reaction monitoring.
