# Supplementary material for: Compound to Extract to Formulation: a knowledge-transmitting approach for metabolites identification of Gegen-Qinlian Decoction, a traditional Chinese medicine formula
Source: Sci Rep. 2016 Dec 20;6:39534. doi: 10.1038/srep39534 (PMC5171860; doi:10.1038/srep39534)
Supplement: Supporting Information [file srep39534-s1.pdf]

Supplementary Information

Compound to Extract to Formulation: a knowledge-transmitting approach for metabolites identification of Gegen-Qinlian Decoction, a traditional Chinese medicine formula

Xue Qiao, Qi Wang, Shuang Wang, Wen-juan Miao, Yan-jiao Li, Cheng Xiang, De-an Guo, and Min Ye*

Affiliation:

State Key Laboratory of Natural and Biomimetic Drugs, School of Pharmaceutical Sciences, Peking University, 38 Xueyuan Road, Beijing 100191, China

Correspondence

State Key Laboratory of Natural and Biomimetic Drugs, School of Pharmaceutical Sciences, Peking University, 38 Xueyuan Road, Beijing 100191, China. Email address: yemin@bjmu.edu.cn (M. Ye). Tel.: +86 10 82801516. Fax: +86 10 82802024.

Table of content

Fig. 1S. Metabolic pathway of **C2** in rats.

Table 1S. Metabolites identified from single *Pueraria* compounds.

Table 2S. Metabolites identified from single *Scutellaria* compounds.

Table 3S. Metabolites identified from single *Coptidis* alkaloids.

Table 4S. Metabolites of Scutellariae Radix extract.

Table 5S. Instrument parameters for neutral loss scan and precursor ion scan.

Table 6S. Metabolites of Puerariae Lobatae Radix extract.

Table 7S. Metabolites of Coptidis Rhizoma extract.

Table 8S.Instrment parameters for SRM scans.

Table 9S. Source herb and *in vivo* distribution of GQD metabolites.

Fig. 1S.

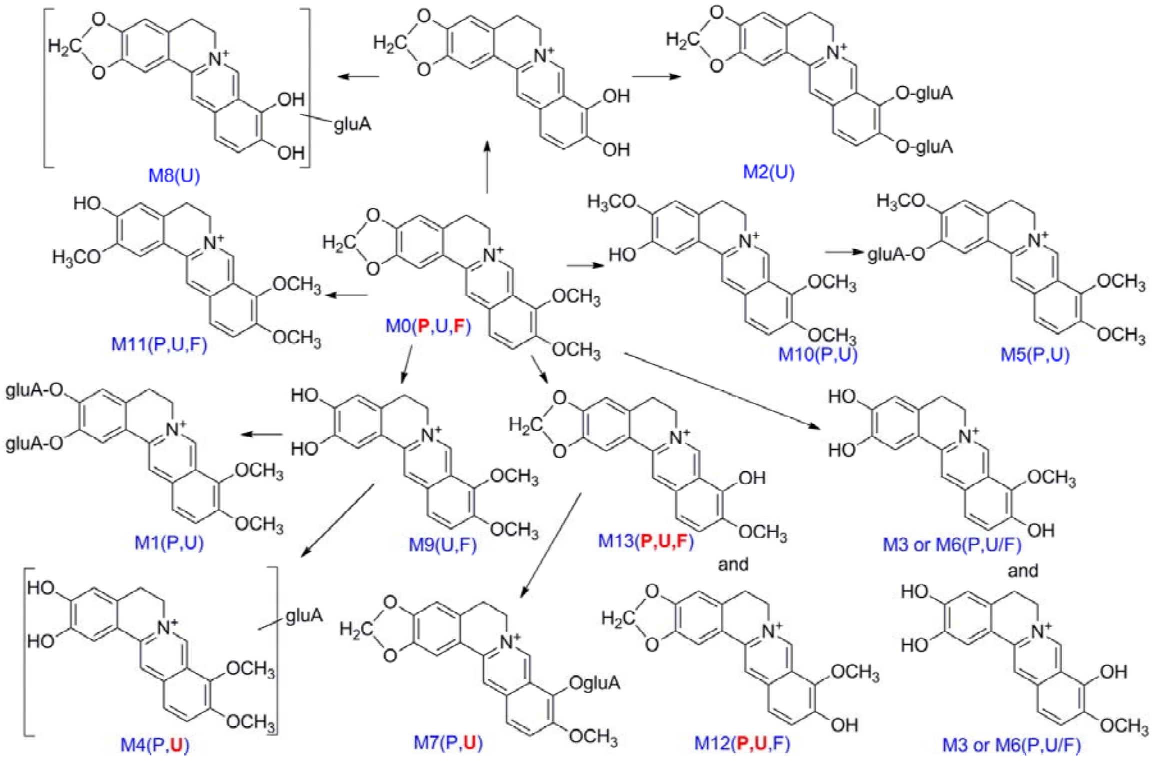
M8(U)
M2(U)
M11(P,U,F)
M0(P,U,F)
M10(P,U)
M5(P,U)
M1(P,U)
M9(U,F)
M13(P,U,F)
M3 or M6(P,U/F)
and
and
M4(P,U)
M7(P,U)
M12(P,U,F)
M3 or M6(P,U/F)

Table 1S. Metabolites identified from single *Pueraria* compounds.

No.	t_R (min)	Identification	*m/z*	UV	(-)-ESI-MS^n	Formula	Source compd. and distribution in biosamples		
							Plasma	Urine	Feces
PS1	9.0	Δ*3'-hydroxypuerarin-*O*-gluA	607	ND	ND	$C_{27}H_{28}O_{16}$	P10	P2,P10	-
PS2	9.3	*3'-hydroxypuerarin	431	ND	MS2:311; MS3:255,283	$C_{21}H_{20}O_{10}$	P2	P2,P10	P2,P10
PS3	9.3	Δ*puerarin-*O*-gluA	591	252,300	MS2:175,**415**; MS3:267,295,325	$C_{27}H_{28}O_{15}$	P9	P9	-
PS4	9.5	Δ*3'-methoxypuerarin-*O*-gluA	621	242,308	MS2:445; MS3:297,325,355	$C_{28}H_{30}O_{16}$	P2,P10	P2,P10	-
PS5	9.6	Δ*3'-methoxypuerarin-*O*-Sul	525	244,308	MS2:445; MS3:430,325,355	$C_{22}H_{22}O_{13}S$	P2	P2,P10	-
PS6	9.6	Δ*puerarin-*O*-Sul	495	244,306	MS2:**325**,375,405,415; MS3:267,307	$C_{21}H_{20}O_{12}S$	P9	P9	-
PS7	9.7	Δ*3'-hydroxypuerarin-*O*-Sul	511	ND	MS2:341,391,431	$C_{21}H_{20}O_{13}S$	P10	P10	-
PS8	9.8	Δ*daidzein-*O*-Sul-*O*-gluA	509	256,306	MS2:253,**333**; MS3:253	$C_{21}H_{18}O_{13}S$	P1,P3,P9	-	-
PS9	10.3	*mirificin	547	268,365	MS2:267,**295**,325; MS3:267	$C_{26}H_{28}O_{13}$	P6	P6	P6
PS10	10.5	*puerarin	415	250,306	MS2:267,**295**; MS3:267	$C_{21}H_{20}O_{9}$	P9	P9	P9
PS11	10.6	*3'-methoxypuerarin	445	250,294	MS2:297,**325**; MS3:297,310	$C_{22}H_{22}O_{10}$	P2,P10	P2,P10	P2,P10
PS12	11.7	Δ*daidzein-*O*-gluA	429	258	MS2:175,**253**; MS3:209,225	$C_{21}H_{18}O_{10}$	P1,P3,P9	P1,P3,P9	P9
PS13	11.9	Δ*equol-*O*-Sul-*O*-gluA	497	ND	ND	$C_{21}H_{22}O_{12}S$	P1,P3,P9	-	-
PS14	12.6	Δ*4'-methoxypuerarin-7-*O*-gulA	605	ND	MS2:309,339,**429**; MS3:281,309	$C_{28}H_{30}O_{15}$	P6	P6	-
PS15	13.5	*formononetin 8-C-glu(6-1)-api	561	250,308	MS2:**309**,339; MS3:281	$C_{27}H_{30}O_{13}$	P6	P6	P6
PS16	14.3	*4'-methoxypuerarin	429	230,324	MS2:281,**309**,339; MS3:281	$C_{22}H_{22}O_{9}$	P6	P6	P6
PS17	15.0	Δ*equol-*O*-gluA	417	224,280	MS2:175,241	$C_{21}H_{22}O_{9}$	P1,P3,P9	P1,P3,P9	-
PS18	16.0	Δ*daidzein-*O*-Sul	333	250,304	MS2:225,**253**; MS3:197,225	$C_{15}H_{10}O_{7}S$	P1,P3,P9	P1,P3,P9	P1,P3
PS19	16.8	3'-hydroxydaidzein	269	ND	MS2:225,241	$C_{15}H_{10}O_{5}$	-	P2,P10	P2
PS20	18.4	Δ**O*-desmethylangolensin-*O*-gluA	433	272,320	MS2:175,**257**; MS3:109,147,163,239	$C_{21}H_{22}O_{10}$	P1,P3	P1,P3	-
PS21	18.8	*3'-methoxydaidzein	283	248,286	MS2:268	$C_{16}H_{12}O_{5}$	-	P1,P2,P3,P10	P1,P2,P3
PS22	18.9	*daidzein	283	250,288	MS2:268; MS3:135,161,195,211,224,240	$C_{15}H_{10}O_{4}$	P9	P1,P3,P6,P9	P1,P3,P6,P9
PS23	18.9	Δ*equol-*O*-Sul	321	220,282	MS2:241; MS3:109,121	$C_{15}H_{14}O_{6}S$	P1,P3,P9	P1,P3,P9	P1,P3
PS24	19.3	Δ**O*-desmethylangolensin-*O*-Sul	337	224,284	MS2:257; MS3:135	$C_{15}H_{14}O_{7}S$	-	P1,P3	-
PS25	19.6	Δ*equol-*O*-Sul	321	220,282	MS2:241	$C_{15}H_{14}O_{6}S$	-	P1,P3	P1,P3
PS26	20.6	*equol	241	ND	ND	$C_{15}H_{14}O_{3}$	-	P1,P3,P9	P1,P3,P9
PS27	21.0	**O*-desmethylangolensin	257	278,314	MS2:109,147	$C_{15}H_{14}O_{4}$	-	P1,P3	P1,P3
PS28	21.5	*formononetin	267	250,300	MS2:252	$C_{16}H_{12}O_{4}$	-	P1,P3, P6	P1,P3, P6

Table 2S. Metabolites identified from single *Scutellaria* compounds.

No.	t_R (min)	Identification	*m/z*	UV	(-)-ESI-MS^n	Formula	Source compd. and distribution in biosamples Plasma	Urine	Feces
SS1	8.3	Δ*pentahydroxyflavanone-O-gluA	479	226,286,326	MS2:175,275,303,451; MS3:125,177	$C_{21}H_{20}O_{13}$	S1	S1	-
SS2	9.0	Δ*pentahydroxyflavanone-O-gluA	479	224,284	MS2:303,451; MS3:125	$C_{21}H_{20}O_{13}$	S1	S1	-
SS3	9.6	Δ*pentahydroxyflavonol-O-gluA	477	220,250,290	MS2:301	$C_{21}H_{18}O_{13}$	S1	S1	-
SS4	10.0	Δ*pentahydroxyflavanone-O-gluA	479	226,288	MS2:285,303,353; MS3:125,177	$C_{21}H_{20}O_{13}$	S1	S1	-
SS5	10.7	Δ*pentahydroxyflavanone-O-Sul	383	224,284,344	MS2:303; MS3:125,177,217,285	$C_{15}H_{12}O_{10}S$	-	S1	-
SS6	11.0	Δ*pentahydroxyflavanone-O-Sul	383	222,278,334	MS2:303; MS3:177	$C_{15}H_{12}O_{10}S$	-	S1	-
SS7	12.2	*chrysin 6-C-ara-8-C-glc	547	272,324	MS2:337,367,427,457,487,529;	$C_{26}H_{28}O_{13}$	S4	S4	S4
SS8	12.6	*pentahydroxyflavanone	303	226,290	MS2:125,177,259,285	$C_{15}H_{12}O_{7}$	S1	S1	S1
SS9	12.6	Δ*pentahydroxyflavonol-O-Sul	381	ND	MS2:301; MS3:125,147,191	$C_{15}H_{10}O_{10}S$	-	S1	-
SS10	14.3	Δ*baicalein-7-O-glc-O-gluA	607	ND	MS2:431	$C_{27}H_{28}O_{16}$	S10,S16	S10,S16	-
SS11	14.5	Δ*baicalein-di-O-gluA	621	272,310	MS2:269,445; MS3:175,269	$C_{27}H_{26}O_{17}$	S10,S16	S10,S16	-
SS12	14.7	*pentahydroxyflavonol	301	254,302,344	MS2:125,151,193,229,257	$C_{15}H_{10}O_{7}$	-	S1	S1
SS13	15.3	Δ*wogonin-di-O-gluA	635	ND	MS2:459; MS3:283	$C_{28}H_{28}O_{17}$	S2,S15	S2	-
SS14	15.4	Δ*chrysin 6-C-ara-O-gluA	561	272,302	MS2:385; MS3:267,295,325	$C_{26}H_{26}O_{14}$	S4	-	-
SS15	16.0	*chrysin 6-C-ara	385	274,316	MS2:295,325; MS3:267	$C_{20}H_{18}O_{8}$	S4	-	S4
SS16	17.5	Δ*baicalin	445	272,310	MS2:269; MS3:251	$C_{21}H_{18}O_{11}$	S10,S14,S16	S14,S10,S16	S10,S16
SS17	18.6	Δ*wogonin-5-O-gluA	459	222,266	MS2:175,268,283; MS3:137,239,268	$C_{22}H_{20}O_{11}$	S2,S15	S2,S15	-
SS18	18.6	Δ norwogonin-O-gluA	445	224,274	MS2:175,269	$C_{21}H_{18}O_{11}$	S15	S2,S15	-
SS19	18.7	Δ* oroxylin A-7-O-gluA	459	262,324	MS2:175,283; MS3:268	$C_{22}H_{20}O_{11}$	S10,S14,S16	S14,S10,S16	-
SS20	18.9	Δ norwogonin-O-gluA	445	276	MS2:175,269; MS3:197,226,241	$C_{21}H_{18}O_{11}$	S2,S15	S2,S15	-
SS21	19.0	baicalein-O-glc	431	ND	MS2:197,269	$C_{21}H_{20}O_{10}$	S16	S10,S16	-
SS22	19.0	Δ*baicalein-O-gluA	445	270,316	MS2:175,269	$C_{21}H_{18}O_{11}$	S10,S14,S16	S10,S16	-
SS23	19.3	norwogonin-O-gluA	445	ND	ND	$C_{21}H_{18}O_{11}$	S2,S15	-	-
SS24	19.5	Δ*wogonoside	459	224,274,344	MS2:175,283; MS3:137,268	$C_{22}H_{20}O_{11}$	S2,S15	S2,S15	S15
SS25	21.3	*baicalein	269	278,324	MS2:225,251	$C_{15}H_{10}O_{5}$	S10,S16	S10,S16	S10,S16
SS26	23.5	*wogonin	283	276	MS2:109,137,197,268	$C_{16}H_{12}O_{5}$	S2,S15	S2,S15	S2,S15

SS27	23.8	*oroxylin A	283	270,320	MS2:137,191,268	$C_{16}H_{12}O_5$	S10	S14,S10,S16	S14

Table3S. Metabolites identified from single *Coptidis* alkaloids.

No.	t_R (min)	Identification	*m/z*	UV	(-)-ESI-MS^n	Formula	Source compd. and distribution in biosamples		
							Plasma	Urine	Feces
CS1	9.8	demethyleneberberine-di-*O*-gluA	676	272,334	MS2:324,**500**; MS3:324,309	$C_{31}H_{33}NO_{16}$	C2	C2	-
CS2	10.4	didemethylberberine-di-*O*-gluA	660	272,344	MS2:308,**484**; MS3:308	$C_{30}H_{29}NO_{16}$	-	C2	-
CS3	11.3	tridemethylated palmatine	310	ND	MS2:295	$C_{18}H_{15}NO_4$	C2,C1	C1	C2
CS4	11.5	Δdemethyleneberberine-*O*-gluA	500	278,344	MS2:**324**; MS3:280,309	$C_{25}H_{25}NO_{10}$	C2,C1	C2,C1	-
CS5	11.6	Δ3-methoxydemethyleneberberine-2-*O*-gluA	514	ND	MS2:**338**; MS3:294,323	$C_{26}H_{27}NO_{10}$	C2,C1	C2,C1	-
CS6	11.7	tridemethylated palmatine	310	ND	MS2:295	$C_{18}H_{15}NO_4$	C2	C1	C2
CS7	11.9	Δmonohydroxypalmatine-*O*-gluA	514	ND	ND	$C_{26}H_{27}NO_{10}$	C1	C1	-
CS8	12.2	Δberberrubine-9-*O*-gluA	498	236,262,346	MS2:**322**; MS3:307	$C_{25}H_{23}NO_{10}$	C2	C2	-
CS9	12.6	Δdidemethylberberine-*O*-gluA	484	ND	MS2:**308**; MS3:251,261,280,295	$C_{24}H_{21}NO_{10}$	-	C2	-
CS10	13.3	demethyleneberberine	324	ND	MS2:280,**309**; MS3:280	$C_{19}H_{17}NO_4$	C1	C2,C1	C2
CS11	15.6	3-methoxydemethyleneberberine	338	230,345	MS2:**323**; MS3:294,322,307	$C_{20}H_{19}NO_4$	C2,C1	C2,C1	C2
CS12	16.2	*jatrorrhizine	338	264,342	MS2:294,**323**; MS3:294,322	$C_{20}H_{19}NO_4$	C2	C2	-
CS13	16.5	thalifendine	322	ND	MS2:**307**; MS3:279,292	$C_{19}H_{15}NO_4$	C2	C2	C2
CS14	16.8	monohydroxypalmatine	338	ND	ND	$C_{20}H_{19}NO_4$	C1	C1	C1
CS15	17.5	berberrubine	322	275,330	MS2:**307**; MS3:279	$C_{19}H_{15}NO_4$	C2	C2	C2
CS16	18	*palmatine	352	270,350	MS2:**337**; MS3:308,336	$C_{21}H_{21}NO_4$	C1	C1	C1
CS17	18.3	*berberine	336	234,264,346	MS2:292,**321**; MS3:292,318,320	$C_{20}H_{17}NO_4$	C2	C2	C2

Table 4S. Metabolites of Puerariae Lobatae Radix extract.

No.	t_R (min)	*m/z*	NL	PR	SRM	UV (nm)	Formula	HRMS, $[M-H]^-$			Identification	P	U	F
								Measured	Predicted	Δ(ppm)				
PE1	11.7	591	176	415	**591/415**	242	$C_{27}H_{28}O_{15}$	591.1356	591.1355	-0.09	Δ*puerarin-O-gluA	DH,DL	DH,DL	/
PE2	11.9	607	176	431	**607/431**	nd	$C_{27}H_{28}O_{16}$	607.1301	607.1305	0.59	Δ*3'-hydroxypuerarin-O-gluA	DH,DL	DH,DL	/
PE3	12.5	431	120	311	**431/311**	222,278	$C_{21}H_{20}O_{10}$	431.0988	431.0984	-0.99	*3'-hydroxypuerarin	/	DH,DL	DH
PE4	12.8	621	176	445	**621/445**	250	$C_{28}H_{30}O_{16}$	621.1460	621.1461	0.17	Δ*3'-methoxypuerarin-O-gluA	DH	DH,DL	/
PE5	13.8	495	80	415	**495/415**	224,300	$C_{21}H_{20}O_{12}S$	495.0602	495.0603	0.14	Δ*puerarin-O-Sul	DH,DL	DH,DL	/
PE6	14.0	525	80	445,325	**525/325,525/445**	224,308	$C_{22}H_{22}O_{13}S$	525.0692	525.0708	3.11	Δ*3'-methoxypuerarin-O-Sul	DH	DH,DL	/
PE7	14.1	511	80	431	**511/431**	nd	$C_{21}H_{20}O_{13}S$	511.0449	511.0552	0.56	Δ*3'-hydroxypuerarin-O-Sul	DH,DL	DH,DL	/
PE8	15.6	415	120	295	**415/295**	256,304	$C_{21}H_{20}O_{9}$	415.1033	415.1035	0.37	*puerarin	DH,DL	DH,DL	DH
PE9	16.6	509	176,80(nd)	253,333	**509/429,509/333**	256,306	$C_{21}H_{18}O_{13}S$	547.1459	509.0395	-0.34	Δ*daidzein-O-Sul-O-gluA	DH,DL	DH,DL	/
PE10	16.9	547	252	295	**547/295**	250,294	$C_{26}H_{28}O_{13}$	445.1141	547.1457	-0.18	*mirificin	DH,DL	DH,DL	DH
PE11	17.0	445	120	325,430	**445/325,445/430**	250,294	$C_{22}H_{22}O_{10}$	509.0393	445.1140	0.46	*3'-methoxypuerarin	DH,DL	DH,DL	DH
PE12	17.1	525	80	445,325	**525/325,525/445**	nd	$C_{22}H_{22}O_{13}S$	525.0705	525.0708	0.64	methoxypuerarin-O-Sul	DH	DH	/
PE13	17.7	577	252	325	**577/325**	246,344	$C_{27}H_{30}O_{14}$	577.1562	577.1563	0.14	*3'-methoxymirificin	DH,DL	DH,DL	DH
PE14	19.5	445	120	325,430	**445/325,445/430**	nd	$C_{22}H_{22}O_{10}$	445.1142	445.1140	-0.4	methoxypuerarin	DH,DL	DH,DL	/
PE15	20.6	429	176	253	**429/253**	260,298	$C_{21}H_{18}O_{10}$	429.0830	429.0827	-0.65	Δ*daidzein-O-gluA	DH,DL	DH,DL	/
PE16	25.8	473	176	297	**473/297**	nd	$C_{23}H_{22}O_{11}$	473.1094	473.1089	-0.98	Δpuerol A-O-gluA	DH	DH	/
PE17	28.5	605	176	429,309	**605/429,605/309**	nd	$C_{28}H_{30}O_{15}$	605.1539	605.1512	-4.46	Δ*4'-methoxypuerarin-7-O-gulA	DH,DL	DH	/
PE18	30.8	497	80,176	321,417	**497/241,497/417**	nd	$C_{21}H_{22}O_{12}S$	497.0765	497.0759	-1.16	Δ*equol-O-Sul-O-gluA	DH,DL	/	/
PE19	31.9	497	80,176	321	**497/241,497/417**		$C_{21}H_{22}O_{12}S$		497.0759		Δ*equol-O-Sul-O-gluA	DH,DL	/	/
PE20	32.0	473	176	297	**473/297**	nd	$C_{23}H_{22}O_{11}$	473.1083	473.1089	1.34	Δpuerol A-O-gluA	DH,DL	DH,DL	/
PE21	32.0	445	176	269	**445/269**	nd	$C_{21}H_{18}O_{11}$	445.0771	445.0776	1.2	Δ3'-hydroxydaidzein-O-gluA	DH,DL	DH,DL	/
PE22	35.3	429			**429/309**	nd	$C_{22}H_{22}O_{9}$	429.1193	429.1191	-0.45	*4'-methoxypuerarin	DH,DL	DH,DL	DH
PE23	35.8	561	252	309	**561/309,561/339**	nd	$C_{27}H_{30}O_{13}$	561.1632	561.1614	-3.26	*formononetin 8-C-glu(6-1)-api	DH,DL	DH,DL	DH
PE24	36.8	497	80	321	**497/241,497/417**		$C_{21}H_{22}O_{12}S$		497.0759		Δ*equol-O-Sul-O-gluA	DH,DL	/	/
PE25	40.2	459	176	283	**459/283**	nd	$C_{22}H_{20}O_{11}$	459.0934	459.0933	-0.25	Δ*3'-methoxydaidzein-O-gluA	DH,DL	DH,DL	/
PE26	40.7	269	28	241	**269/241**	nd	$C_{15}H_{10}O_{5}$	269.0453	269.0455	0.91	3'-hydroxydaidzein	/	DH	/

PE27	41.1	417	176	241	**417/241**	nd	$C_{21}H_{22}O_9$	417.1193	417.1191	-0.46	Δ*equol-O-gluA	DH,DL	DH,DL	/
PE28	43.6	377	80	297	**377/297**	nd	$C_{17}H_{14}O_8S$	377.0352	377.0337	-4.07	Δpuerol A-O- Sul	/	DH,DL	/
PE29	44.5	333	80	253,225	**333/253,333/225**	250,304	$C_{15}H_{10}O_7S$	333.0078	333.0074	-1.06	Δ*daidzein-O-Sul	DH,DL	DH,DL	/
PE30	45.3	363	80	283	**363/283**	220,278	$C_{16}H_{12}O_8S$	363.0181	363.0180	-0.24	Δ*3'-methoxydaidzein-O-Sul	DH,DL	DH,DL	/
PE31	45.8	363	80	283	**363/283**	288,324	$C_{16}H_{12}O_8S$	363.0177	363.0180	0.86	Δ*3'-methoxydaidzein-O-Sul	DH,DL	DH,DL	/
PE32	50.4	297			**297/253**	225,324	$C_{17}H_{14}O_5$	297.0767	297.0768	0.49	puerol A	DH,DL	DH,DL	/
PE33	53.2	443	176	267	**443/267**	nd	$C_{22}H_{20}O_{10}$	443.0984	443.0984	-0.07	*formononetin-7-O-gluA	DH,DL	DH,DL	/
PE34	53.4	487	176	311	**487/311**	nd	$C_{24}H_{24}O_{11}$	487.1250	487.1246	-0.85	Δpuerol B-O-gluA	DH,DL	DH,DL	/
PE35	55.9	255		119,149	**255/119,255/149**	278,314	$C_{15}H_{12}O_4$	255.0662	255.0663	0.32	*dihydrodaidzein	DH	DH	/
PE36	56.0	487	176	311	**487/311**	nd	$C_{24}H_{24}O_{11}$	487.1250	487.1246	-0.85	Δpuerol B-O-gluA	DH,DL	DH,DL	DH
PE37	56.3	253	28	225	**253/225**	260,304	$C_{15}H_{10}O_4$	253.0507	253.0506	-0.27	*daidzein	DH,DL	DH,DL	DH
PE38	57.0	433	176	257	**433/257**	nd	$C_{21}H_{22}O_{10}$	433.1143	433.1140	-0.64	Δ*O-desmethylangolensin-O-gluA	DH,DL	DH,DL	/
PE39	57.1	283	15	268	**283/268**	248,286	$C_{16}H_{12}O_5$	283.0611	283.0612	0.34	*3'-methoxydaidzein	DH,DL	DH,DL	/
PE40	58.5	337	80	257	**337/257**	226,308	$C_{15}H_{14}O_7S$	337.0931	337.0387	-1.04	Δ*O-desmethylangolensin-O-Sul	DH,DL	DH,DL	/
PE41	58.7	321	80	241	**321/241**	nd	$C_{15}H_{14}O_6S$	321.0439	321.0438	0.1	Δ*equol-O-Sul	DH,DL	DH,DL	/
PE42	60.3	321	80	241	**321/241**	220,282	$C_{15}H_{14}O_6S$	321.0440	321.0438	-0.52	Δ*equol-O-Sul	DH,DL	DH,DL	/
PE43	62.0	391	80	311	**391/311**	224,300	$C_{18}H_{16}O_8S$	391.0492	391.0493	0.29	Δpuerol B-O- Sul	DH,DL	DH,DL	/
PE44	62.6	241		119	**241/119**		$C_{15}H_{14}O_3$	241.0873	241.0870	-1.17	*equol	/	DH	/
PE45	63.3	311		119	**311/119,311/267**	224,324	$C_{18}H_{16}O_5$	311.0926	311.0925	-0.33	puerol B	DH	DH,DL	DH
PE46	65.4	257		135	**257/135,257/109**	278,314	$C_{15}H_{14}O_4$	257.0819	257.0819	0.13	*O-desmethylangolensin	/	DH,DL	DH
PE47	66.2	267	15		**267/252**	250,300	$C_{16}H_{12}O_4$	267.0662	267.0663	0.31	*formononetin	DH	DH,DL	DH

Table 5S. Instrument parameters for neutral loss scan and precursor ion scan.

Ion mode	Negative	Positive
Spray voltage	3800	4000
Sheath gas pressure	50	45
Aux gas pressure	5	5
Capillary temperature	330	330
Capillary offset	-35	35
Source framentation voltage	5-10	0
Collion energy	25-30	25-30

Table 6S. Metabolites of Scutellariae Radix extract.

No.	t_R	*m/z*	NL	PR	SRM	UV (nm)	Formula	HRMS, $[M-H]^-$			Identification	P	U	F
	(min)							Measured	Predicted	Δ(ppm)				
SE1	8.5	479	176	303, 125	479/303,**479/125**	286, 326	$C_{21}H_{20}O_{13}$	479.0842	479.0831	-2.26	Δ*pentahydroxyflavanone-*O*-gluA	DH,DL	DH,DL	/
SE2	9.4	479	176	303, 125	479/303,**479/125**	224, 284	$C_{21}H_{20}O_{13}$	479.0847	479.0831	-3.3	Δ*pentahydroxyflavanone-*O*-gluA	DH,DL	DH,DL	/
SE3	11.5	477	176	301, 151	**477/301**,477/125	250, 290	$C_{21}H_{18}O_{13}$	477.0686	477.0675	-2.38	Δ*pentahydroxyflavonol-*O*-gluA	/	DH,DL	/
SE4	12.9	479	176	303, 125	479/303,**479/125**	226, 288	$C_{21}H_{20}O_{13}$	479.0845	479.0831	-2.89	Δ*pentahydroxyflavanone-*O*-gluA	/	DH,DL	/
SE5	15.6	383	80	303, 177	383/303	284, 344	$C_{15}H_{12}O_{10}S$	383.008	383.0078	-0.41	Δ*pentahydroxyflavanone-*O*-Sul	/	DH	/
SE6	16.8	477	176	301, 151	**477/301**,477/125	250, 290	$C_{21}H_{18}O_{13}$	477.0696	477.0675	-4.47	Δ*pentahydroxyflavonol-*O*-gluA	/	DH,DL	/
SE7	20.3	383	80	303, 177	383/303	278, 334	$C_{15}H_{12}O_{10}S$	383.0089	383.0078	-2.76	Δ*pentahydroxyflavanone-*O*-Sul	/	DH	/
SE8	20.6	303	18	125, 177	**303/125**,303/177	226, 290	$C_{15}H_{12}O_7$	303.0511	303.051	-0.24	*pentahydroxyflavanone	DH	DH,DL	DH
SE9	21.8	381	80	301	381/301,381/175	nd	$C_{15}H_{10}O_{10}S$	380.9927	380.9922	-1.33	Δ*pentahydroxyflavonol-*O*-Sul	/	DH,DL	/
SE10	22.6	601	176	345, 425	**601/425**,601/345	nd	$C_{23}H_{22}O_{17}S$	601.0514	601.0505	-1.51	Δviscidulin III-*O*-gluA-*O*-Sul	DH,DL	DH,DL	/
SE11	23.0	577	120	427	577/457	nd	$C_{27}H_{30}O_{14}$	577.1563	577.1566	-0.15	chrysin 6,8-di-*C*-glucoside	DH,DL	DH,DL	DH
SE12	25.8	521	176	345, 330	521/345	200, 264	$C_{23}H_{22}O_{14}$	521.0949	521.0937	-2.34	Δviscidulin III-*O*-gluA	/	DH,DL	/
SE13	27.6	301	176	125, 175	**301/125**,301/175	302, 344	$C_{15}H_{10}O_7$	301.0359	301.0354	-1.73	*pentahydroxyflavonol	/	DH,DL	DH
SE14	28.2	547	120, 90	427, 295, 457	547/427,547/457	272, 324	$C_{26}H_{28}O_{13}$	547.1478	547.1457	-3.8	*chrysin 6-*C*-ara-8-*C*-glu	DH,DL	DH,DL	DH
SE15	29.5	547	120, 90	427, 295, 457	547/427,547/457	272, 324	$C_{26}H_{28}O_{13}$	547.1481	547.1457	-4.35	isomer of chrysin 6-*C*-ara-8-*C*-glu	DH,DL	DH,DL	DH
SE16	31.4	521	176	345, 330	521/345	212, 268	$C_{23}H_{22}O_{14}$	521.0939	521.0937	-0.42	Δviscidulin III-*O*-gluA	DH,DL	DH	/
SE17	31.6	461	176	285	461/285	280, 335	$C_{21}H_{18}O_{12}$	461.0732	461.0725	-1.41	* scutellarin	/	DH	/
SE18	32.0	605	176	253,429	605/253,605/429		$C_{27}H_{26}O_{16}$	605.1166	605.1148	-2.96	Δ*chrysin-di-*O*-gluA	DH,DL	DH,DL	/
SE19	32.5	547	120, 90	427, 295, 457	547/427,547/457	274, 314	$C_{26}H_{28}O_{13}$	547.1482	547.1457	-4.53	isomer of chrysin 6-*C*-ara-8-*C*-glu	DH,DL	DH,DL	DH
SE20	34.8	547	120, 90	427, 295, 457	547/427,547/457	274, 324	$C_{26}H_{28}O_{13}$	547.1478	547.1457	-3.8	isomer of chrysin 6-C-ara-8-C-glu	DH	DH,DL	DH
SE21	39.4	607	176	269	**607/431**,607/269	272, 312	$C_{27}H_{28}O_{16}$	607.1309	607.1305	-0.73	Δ*baicalein-7-*O*-glu-*O*-gluA	DH,DL	DH,DL	/
SE22	40.1	621	176	269	**621/445**,621/269	272, 310	$C_{27}H_{26}O_{17}$	621.112	621.1097	-3.66	Δ*baicalein-di-*O*-gluA	DH,DL	DH,DL	/
SE23	43.5	635	176	459	**635/459**,635/283	266, 308	$C_{28}H_{28}O_{17}$	635.1284	635.1254	-4.76	Δ*wogonin-di-*O*-gluA	DH,DL	DH,DL	/
SE24	43.6	425	80	345, 330	425/345	224, 264	$C_{17}H_{14}O_{11}S$	425.0194	425.0184	-2.33	Δviscidulin III-*O*-Sul	DH,DL	DH,DL	/
SE25	44.0	577	176	401	577/401	nd	$C_{26}H_{26}O_{15}$	577.1221	577.1199	-3.82	Δapigenin-8-*C*-ara-*O*-gluA	/	DH,DL	/
SE26	44.1	561	176	385, 295, 325	561/385	272, 302	$C_{26}H_{26}O_{14}$	561.1277	561.125	-4.84	Δ*chrysin 6-*C*-ara-*O*-gluA	DH	DH	/
SE27	46.7	651	176	299, 475	651/475,651/299	278, 318	$C_{28}H_{28}O_{18}$	651.1228	651.1203	-3.85	Δ*lateriflorein 7-*O*-gluA-*O*-gluA	DH,DL	DH,DL	/
SE28	50.7	345	15	330, 297	**345/315**,345/330	222, 264	$C_{17}H_{14}O_8$	345.0626	345.0616	-2.92	viscidulin III	/	DH,DL	DH

SE29	54.2	445	176	269	445/269	272, 310	$C_{21}H_{18}O_{11}$	445.0797	445.0776	-4.63	Δ*baicalin	DH,DL	DH,DL	DH
SE30	54.6	525	80	445, 269	525/269,525/445,	nd	$C_{21}H_{18}O_{14}S$	525.0368	525.0344	-4.47	Δnorwogonin-*O*-Sul	DH,DL	DH,DL	DH
SE31	55.5	539	80		539/283	nd	$C_{22}H_{20}O_{14}S$	539.0525	539.0501	-4.44	Δwogonin-*O*-gluA-*O*-Sul	DH	DH	/
SE32	56.3	509	80, 176	253	509/429,509/333,	nd	$C_{21}H_{18}O_{13}S$	509.0415	509.0395	-3.85	Δchrysin-*O*-gluA-*O*-Sul	DH	DH	/
SE33	57.0	385	90	295	385/295	274, 316	$C_{20}H_{18}O_{8}$	385.0945	385.0929	-4.17	*chrysin 6-*C*-ara	DH	DH	DH
SE34	57.2	459	176	283, 268	459/283	222, 266	$C_{22}H_{20}O_{11}$	459.0941	459.0933	-1.77	Δ*wogonin-5-*O*-gluA	DH,DL	DH,DL	/
SE35	57.7	555	80	475, 299	**555/475**,555/299	nd	$C_{22}H_{20}O_{15}S$	555.0474	555.045	-4.29	Δ*lateriflorein 7-*O*-gluA-*O*-Sul	DH,DL	DH,DL	/
SE36	57.8	525	80, 176	269, 349	525/269	nd	$C_{21}H_{18}O_{14}S$	525.0363	525.0344	-3.52	Δnorwogonin-*O*-Sul	DH,DL	/	/
SE37	57.9	445	176	269	445/269	224, 274	$C_{21}H_{18}O_{11}$	445.0787	445.0776	-3.73	Δ norwogonin-*O*-gluA	/	DH,DL	/
SE38	58.0	429	176	253	429/253	nd	$C_{21}H_{18}O_{10}$	429.083	429.0827	-0.65	Δ*chrysin-*O*-gluA	DH	DH	/
SE39	58.0	539	80	283	539/283	222, 274	$C_{22}H_{20}O_{14}S$	539.0525	539.0501	-4.44	Δ*oroxylin A-7-*O*-gluA-*O*-Sul	DH,DL	DH,DL	/
SE40	58.4	511	80,	269, 431	511/431	272, 310	$C_{21}H_{20}O_{13}S$	511.0576	511.0552	-4.72	Δbaicalein-*O*-glu-*O*-Sul	DH,DL	DH,DL	/
SE41	58.8	445	176	269	445/269	226, 270	$C_{21}H_{18}O_{11}$	445.0787	445.0776	-2.39	Δnorwogonin-*O*-gluA	DH,DL	DH,DL	/
SE42	58.8	429	176	253	429/253	nd	$C_{21}H_{18}O_{10}$	429.0832	429.0827	-1.12	Δ*chrysin-7-*O*-gluA	DH,DL	DH	DH
SE43	58.9	525	80		525/269	nd	$C_{21}H_{18}O_{14}S$	525.0367	525.0344	-4.28	Δ*baicalein-*O*-gluA	DH,DL	DH,DL	/
SE44	59.1	425	80	345, 330	425/345	224, 264	$C_{17}H_{14}O_{11}S$	425.0198	425.0184	-3.27	Δviscidulin III-*O*-Sul	DH	DH,DL	/
SE45	59.2	459	176	283	459/283	262, 324	$C_{22}H_{20}O_{11}$	459.0928	459.0933	1.05	Δ* oroxylin A-7-*O*-gluA	DH,DL	DH,DL	/
SE46	59.4	475	176	299	475/299	278, 318	$C_{22}H_{20}O_{12}$	475.0883	475.0882	-0.21	Δ*lateriflorein 7-*O*-gluA-*O*-Sul	DH,DL	DH,DL	/
SE47	59.6	431	162	269	431/269	274, 310	$C_{21}H_{20}O_{10}$	431.0992	431.0984	-1.92	baicalein-*O*-glu	/	DH,DL	/
SE48	59.8	525	80	269	525/269	nd	$C_{21}H_{18}O_{14}S$	525.0361	525.0344	-3.14	Δ*baicalein-*O*-Sul	DH,DL	/	/
SE49	59.9	445	176	269	445/269	270, 316	$C_{21}H_{18}O_{11}$	445.0791	445.0776	-3.28	Δ*baicalein-*O*-gluA	DH,DL	DH,DL	DH
SE50	60.2	481	80	401	481/401		$C_{20}H_{18}O_{12}S$	481.0462	481.0446	-3.28	Δapigenin-8-*C*-ara-*O*-Sul	DH,DL	DH,DL	/
SE51	60.3	459	176	283, 268	459/283	274, 344	$C_{22}H_{20}O_{11}$	459.0949	459.0933	-3.51	Δ*wogonoside	DH,DL	DH	/
SE52	63.9	299	15		299/284	280, 325	$C_{16}H_{12}O_{6}$	299.0567	299.0561	-1.96	lateriflorein	/	DH,DL	/
SE53	64.2	269	28	241	**269/241**,269/251	278, 324	$C_{15}H_{10}O_{5}$	269.0454	269.0455	0.54	*baicalein	DH,DL	DH,DL	DH
SE54	69.6	283	15	268, 119	283/268	276	$C_{16}H_{12}O_{5}$	283.0613	283.0612	-0.36	*wogonin	DH,DL	DH,DL	DH
SE55	69.8	253		101, 151	253/101,**253/151**	255, 290	$C_{15}H_{10}O_{4}$	253.0505	253.0506	0.52	*chrysin	/	DH,DL	DH
SE56	70.7	283	15	268	**283/268**,283/137	270, 320	$C_{16}H_{12}O_{5}$	283.0613	283.0612	-0.36	*oroxylin A	DH,DL	DH,DL	DH

Table 7S. Metabolites of Coptidis Rhizoma extract.

No.	t_R (min)	*m/z*	NL	PR	SRM	UV (nm)	Formula	HRMS, $[M-H]^-$			Identification	P	U	F
								Measured	Predicted	Δ(ppm)				
CE1	8.8	534	176	358	**534/358,534/340**	224,282	$C_{26}H_{31}NO_{11}$	534.1976	534.1970	-1.15	Δmonohydroxymagnoflorine-O-gluA	DH,DL	DH,DL	/
CE2	9.6	534	176	358	**534/358,534/340**	222,280	$C_{26}H_{31}NO_{11}$	534.1982	534.1970	-2.27	Δmonohydroxymagnoflorine-O-gluA	DH,DL	DH,DL	/
CE3	12.8	548	176	372	**548/372,548/354**	256,312	$C_{27}H_{33}NO_{11}$	548.2144	548.2126	-3.22	Δmethoxymagnoflorine-O-gluA	DH,DL	DH,DL	/
CE4	13.2	534	176	358	**534/358,534/340**	224,272	$C_{26}H_{31}NO_{11}$	534.1993	534.1970	-4.34	Δmonohydroxymagnoflorine-O-gluA	DH,DL	DH,DL	/
CE5	14.0	518	176	342	**518/342**	240,325	$C_{26}H_{31}NO_{10}$	518.2034	518.2021	-2.57	Δ*magnoflorine-O-gluA	DH,DL	DH,DL	/
CE6	14.1	676	176	324	**676/500,676/324**	nd	$C_{31}H_{33}NO_{16}$	676.1906	676.1872	-4.87	Δdemethyleneberberine-di-O-gluA	DH,DL	DH,DL	/
CE7	14.4	358	18	340	**358/340,358/297**	260,340	$C_{20}H_{23}NO_{5}$	358.1663	358.1649	-3.92	monohydroxymagnoflorine	/	DH,DL	/
CE8	14.6	484	176	308	**484/308**	240,325	$C_{24}H_{21}NO_{10}$	484.1260	484.1238	-4.51	Δdidemethylepiberberine-O-gluA	DH,DL	DH,DL	/
CE9	15.2	498	176	322	**498/322,487/307**	274,344	$C_{25}H_{23}NO_{10}$	498.1399	498.1395	-0.86	Δ*groenlandicine-3-O-gluA	DH,DL	DH,DL	/
CE10	15.5	342	45	297	**342/297,342/280**	235,270	$C_{20}H_{23}NO_{4}$	342.1714	342.1700	-4.15	*magnoflorine	DH,DL	DH,DL	/
CE11	15.8	660	176	484	**660/484,660/308**	nd	$C_{30}H_{29}NO_{16}$	660.1569	660.1559	-1.5	Δdidemethylberberine-di-O-gluA	DH,DL	DH,DL	/
CE12	18.0	548	176	372	**548/372,548/354**	240,330	$C_{27}H_{33}NO_{11}$	548.2153	548.2126	-4.87	Δmethoxymagnoflorine-O-gluA	DH,DL	DH,DL	/
CE13	18.3	310	15	295	**310/295**	nd	$C_{18}H_{15}NO_{4}$	310.1060	310.1074	4.48	tridemethylated palmatine	DH,DL	DH,DL	DH
CE14	19.6	372	18	354	**372/354**	255,360	$C_{21}H_{25}NO_{5}$	372.1799	372.1805	1.75	methoxymagnoflorine	/	DH	/
CE15	20.9	500	176,15	324,309	**500/324,500/309**	275,340	$C_{25}H_{25}NO_{10}$	500.1576	500.1551	-4.96	Δdemethyleneberberine-O-gluA	DH,DL	DH,DL	/
CE16	21.4	498	176	322	**498/322,487/307**	262,342	$C_{25}H_{23}NO_{10}$	498.1418	498.1395	-4.68	Δthalifendine-10-O-gluA	DH,DL	DH,DL	/
CE17	21.6	514	176,15	338	**514/338,514/323**	275,340	$C_{26}H_{27}NO_{10}$	514.1714	514.1708	-1.22	Δ3-methoxydemethyleneberberine-2-O-gluA	DH,DL	DH,DL	/
CE18	24.9	498	176	322	**498/322,487/307**	272,336	$C_{25}H_{23}NO_{10}$	498.1419	498.1395	-4.88	Δberberrubine-9-O-gluA	DH,DL	DH,DL	/
CE19	25.7	324	15	309	**324/309**	nd	$C_{19}H_{17}NO_{4}$	324.1243	324.1230	-3.92	didemethylated palmatine	DH,DL	DH,DL	/
CE20	26.1	324	15	309	**324/309**	nd	$C_{19}H_{17}NO_{4}$	324.1243	324.1230	-3.92	didemethylated palmatine	DH	DH,DL	/
CE21	27.1	322	15	307	**322/307**	262,340	$C_{19}H_{15}NO_{4}$	322.1087	322.1074	-4.1	groenlandicine	DH,DL	DH,DL	DH
CE22	27.6	484	176	308	**484/308**	240,320	$C_{24}H_{21}NO_{10}$	484.1260	484.1238	-4.51	Δdidemethylberberine-O-gluA	/	DH	/
CE23	27.6	324	15	309	**324/309**	nd	$C_{19}H_{17}NO_{4}$	324.1244	324.1230	-4.23	demethyleneberberine	DH,DL	DH,DL	DH
CE24	36.9	320		292	**320/292**	260,345	$C_{19}H_{13}NO_{4}$	320.0931	320.0917	-4.28	*coptisine	DH,DL	DH,DL	DH
CE25	37.2	338	15	323	**338/323,338/322**	230,345	$C_{20}H_{19}NO_{4}$	338.1401	338.1387	-4.2	3-methoxydemethyleneberberine	DH,DL	DH,DL	DH
CE26	37.2	336	15	321	**336/321**	260,345	$C_{20}H_{17}NO_{4}$	336.1247	336.1230	-4.97	*epiberberine	DH,DL	DH,DL	DH
CE27	37.6	322	15	307	**322/307**	260,345	$C_{19}H_{15}NO_{4}$	322.1088	322.1074	-4.41	thalifendine	DH,DL	DH,DL	DH
CE28	38.7	338	15	323	**338/323**	230,340	$C_{20}H_{19}NO_{4}$	338.1403	338.1387	-4.79	*jatrorrhizine	DH,DL	DH,DL	DH

CE29	43.7	322	15	307	**322/307**	275,330	$C_{19}H_{15}NO_4$	322.1088	322.1074	-4.41	berberrubine	/	DH,DL	DH
CE30	49.5	336	15	321	**336/321**	265,345	$C_{20}H_{17}NO_4$	336.1245	336.1230	-4.37	*berberine	DH,DL	DH,DL	DH
CE31	52.1	352	15	337	**352/337**	270,350	$C_{21}H_{21}NO_4$	352.1559	352.1543	-4.46	*palmatine	DH,DL	DH,DL	DH

Table 8S.Instrment parameters for SRM scans.

No.	SRM	CE	SID
1	479/125	30	5
2	534/358	-35	-5
3	479/125	30	5
4	534/358	-35	-5
5	477/125	30	5
6	591/415	25	5
7	607/431	25	5
8	431/311	30	5
9	548/372	-35	-5
10	621/445	25	5
11	479/125	30	5
12	534/358	-35	-5
13	495/415	25	5
14	525/445	-30	-5
15	518/342	25	5
16	593/417	25	5
17	511/431	25	5
18	484/308	-30	-5
19	342/297	-35	-5
20	415/295	25	5
21	660/308	-35	-5
22	509/333	25	5
23	547/295	30	5
24	445/325	30	5
25	525/325	30	5
26	577/325	30	5
27	548/372	-35	-5
28	445/325	30	5
29	429/253	25	5
30	500/324	-30	-5
31	498/322	-20	-5
32	514/338	-30	-5
33	601/425	30	5
34	577/457	30	5
35	511/335	25	5
36	498/322	-25	-5
37	473/297	25	5
38	521/345	25	5
39	322/307	-35	-5
40	324/309	-35	-5
41	549/255	25	5
42	417/255	30	5
43	431/175	25	5
44	513/337	25	5
45	547/427	25	5
46	605/429	30	5
47	431/175	25	5
48	497/241	30	5
49	521/345	25	5
50	497/241	30	5
51	473/297	25	5
52	445/269	25	5
53	605/253	25	5
54	547/427	25	5
55	429/309	30	5
56	561/309	30	5
57	497/241	30	5
58	320/292	-35	-5
59	338/323	-35	-5
60	336/321	-35	-5
61	322/307	-35	-5
62	338/323	-35	-5
63	607/431	25	5
64	621/445	25	5
65	459/283	25	5
66	417/241	25	5
67	513/337	25	5
68	635/459	25	5
69	377/297	25	5
70	577/401	30	5
71	333/253	25	5
72	561/385	30	5
73	363/283	25	5
74	363/283	25	5
75	336/321	-35	-5
76	297/119	30	5
77	345/315	30	5
78	352/337	-35	-5
79	443/267	25	5
80	487/311	25	5
81	445/269	25	5
82	525/445	25	5
83	433/257	25	5
84	255/119	30	5
85	487/311	25	5
86	253/225	30	5
87	509/253	30	5
88	433/257	25	5
89	385/295	25	5
90	283/268	30	5
91	459/283	25	5
92	555/475	25	5
93	525/269	30	5
94	445/269	25	5
95	429/253	25	5
96	539/283	30	5
97	511/431	25	5
98	337/257	25	5
99	321/241	25	5
100	445/269	25	5
101	447/271	25	5
102	525/269	30	5
103	425/345	25	5
104	459/283	25	5
105	475/299	25	5
106	431/269	25	5
107	705/529	25	5
108	445/269	25	5
109	481/401	25	5
110	459/283	25	5
111	337/257	25	5
112	515/339	25	5
113	391/311	25	5
114	271/151	30	5
115	311/119	30	5
116	337/257	25	5
117	299/284	30	5
118	837/351	30	5
119	269/241	30	5
120	257/151	30	5
121	257/135	30	5
122	821/351	30	5
123	267/252	30	5
124	269/254	30	5
125	347/267	25	5
126	283/268	30	5
127	253/151	30	5
128	367/309	30	5
129	283/268	30	5
130	365/307	30	5
131	469/425	30	5

Table 9S. Source herb and *in vivo* distribution of GQD metabolites.

	Sample	Total	P	S	C	G
High	Plasma	96	39	28	13	17
dosage	Urine	109	38	36	21	15
	Feces	20	8	4	6	2
Total		131	44	42	22	23
Clinical	Plasma	77	34	22	13	8
dosage	Urine	90	36	25	19	11
Total		112	44	31	21	16
